# LncRNA-Dependent Mechanisms of Transforming Growth Factor-β: From Tissue Fibrosis to Cancer Progression

**DOI:** 10.3390/ncrna8030036

**Published:** 2022-05-25

**Authors:** Philip Chiu-Tsun Tang, Ying-Ying Zhang, Jane Siu-Fan Li, Max Kam-Kwan Chan, Jiaoyi Chen, Ying Tang, Yiming Zhou, Dongmei Zhang, Kam-Tong Leung, Ka-Fai To, Sydney Chi-Wai Tang, Hui-Yao Lan, Patrick Ming-Kuen Tang

**Affiliations:** 1 Department of Anatomical and Cellular Pathology, State Key Laboratory of Translational Oncology, The Chinese University of Hong Kong, Hong Kong 999077, China; philtang@link.cuhk.edu.hk (P.C.-T.T.); jane.li@link.cuhk.edu.hk (J.S.-F.L.); maxchankamkwan@link.cuhk.edu.hk (M.K.-K.C.); kfto@cuhk.edu.hk (K.-F.T.); 2 Department of Nephrology, Tongji University School of Medicine, Shanghai 200065, China; idklaa@hotmail.com; 3 Division of Nephrology, Department of Medicine, The University of Hong Kong, Hong Kong 999077, China; jiaoyichen92@gmail.com (J.C.); scwtang@hku.hk (S.C.-W.T.); 4 Department of Nephrology, The Third Affiliated Hospital of Southern Medical University, Guangzhou 510080, China; ty.102@163.com; 5 Guangdong Provincial Key Laboratory of Malignant Tumor Epigenetics and Gene Regulation, Guangdong-Hong Kong Joint Laboratory for RNA Medicine, Sun Yat-sen Memorial Hospital, Sun Yat-sen University, Guangzhou 510120, China; zhouym35@mail.sysu.edu.cn; 6 College of Pharmacy, Jinan University, Guangzhou 510632, China; dmzhang701@jnu.edu.cn; 7 Department of Paediatrics, The Chinese University of Hong Kong, Hong Kong 999077, China; ktleung@cuhk.edu.hk; 8 Department of Medicine and Therapeutics, Li Ka Shing Institute of Health Sciences, The Chinese University of Hong Kong, Hong Kong 999077, China; hylan@cuhk.edu.hk

**Keywords:** long non-coding RNA, fibrosis, transforming growth factor-β, cancer, Smad3, TGF-β

## Abstract

Transforming growth factor-β (TGF-β) is a crucial pathogenic mediator of inflammatory diseases. In tissue fibrosis, TGF-β regulates the pathogenic activity of infiltrated immunocytes and promotes extracellular matrix production via de novo myofibroblast generation and kidney cell activation. In cancer, TGF-β promotes cancer invasion and metastasis by enhancing the stemness and epithelial mesenchymal transition of cancer cells. However, TGF-β is highly pleiotropic in both tissue fibrosis and cancers, and thus, direct targeting of TGF-β may also block its protective anti-inflammatory and tumor-suppressive effects, resulting in undesirable outcomes. Increasing evidence suggests the involvement of long non-coding RNAs (lncRNAs) in TGF-β-driven tissue fibrosis and cancer progression with a high cell-type and disease specificity, serving as an ideal target for therapeutic development. In this review, the mechanism and translational potential of TGF-β-associated lncRNAs in tissue fibrosis and cancer will be discussed.

## 1. Introduction

Long non-coding RNAs (lncRNAs) are transcripts with lengths of over 200 nucleotides that together with short microRNAs (miRNAs), small interfering RNAs (siRNAs), small nucleolar RNAs (snoRNAs), small nuclear RNAs (snRNAs), and PIWI-interacting RNAs (piRNAs) constitute a spectrum of non-coding RNA molecules (ncRNAs) characterized by their gene-regulating functions [1,2,3]. Of these, lncRNAs and miRNAs are two major classes of ncRNAs that participate in the pathogenesis of cancer and fibrotic diseases, as dysregulation of lncRNAs and miRNAs interferes with the control of crucial biological processes, including cell proliferation, apoptosis, and extracellular matrix homeostasis [4,5]. Since their discovery with high-throughput RNA sequencing, various biological functions of lncRNAs have been revealed, suggesting that these RNAs may be a missing piece of a complex gene-regulatory mechanism that supports higher life forms and not junk transcripts left over from evolution [6,7]. Emerging evidence indicates that lncRNAs are important for fine-tuning transcription, contributing to the regulatory network of spatial and temporal gene expression with high specificity. Thus, lncRNAs may represent an optimal target for diagnosing and treating diseases.

Transforming growth factor-β (TGF-β) is a crucial cytokine that drives the pathogenesis and development of chronic inflammatory diseases, particularly chronic kidney disease (CKD) and cancer [8,9,10,11]. TGF-β signaling is highly activated in experimental models and in patient biopsies of CKD associated with enhanced production and deposition of extracellular matrix (ECM) components like collagens and fibronectins, contributing to the disruption of tissue structure and eventually leading to end-stage renal disease (ESRD) with complete loss of function [12,13]. However, fibrotic response in a resolvable manner is an essential mechanism for repairing initial tissue injury. Therapy directly targeting TGF-β may lead to side effects in vital organs [14]. This scenario also applies to cancer in that pleiotropic cytokine TGF-β initially suppresses proliferation and induces apoptosis of cancer cells but promotes immunosuppression, angiogenesis, and cancer cell growth via stromal cells in established tumors [15,16]. Therefore, identifying pathogenic downstream mediators of TGF-β is essential for developing a specific strategy that suppresses the pathogenic activity while preserving the therapeutic and physiological activities of TGF-β. TGF-β induced lncRNAs with high spatial and temporal specificity in the pathogenesis of inflammatory disease, serving as an ideal target for developing targeted TGF-β signaling therapy [1,17,18]. In this review, the mechanism of TGF-β-induced lncRNAs in the pathogenesis of renal fibrosis and cancer will be discussed.

## 2. TGF-β1 Signaling Pathways

The TGF-β superfamily is a group of cytokines with shared properties in biosynthesis, signal transduction, and other functions. It consists of four major subfamilies and a group encompassing various divergent members. TGF-β1-3 are the three distinct isoforms; TGF-β1 lacks a TATAA box in its promoter, in contrast to TGF-β2 and TGF-β3 with TATAA boxes [19]. TGF-β1 is well recognized as a key driver of fibrosis and cancer progression, while the roles of TGF-β2 and TGF-β3 are still largely unclear [20]. TGF-β1 can be directly activated by reactive oxygen species, pH, and proteases in various contexts such as tissue injury, stress, viral infection, carcinogenesis, tissue fibrosis, and inflammation [21,22]. TGF-β1 peptide is expressed and secreted as a nonactive complex with latent TGF-β binding protein that is cleaved to release active TGF-β1 [17] for TGF-β1 receptor type II binding, triggering downstream signaling via TGF-β1 receptor type I kinase (TβRI) [23]. Smad proteins are key players in the canonical pathway, while non-Smad signalings are noncanonical pathways. Smad2/3 activated by TβRI kinase forms a heterotrimeric complex with Smad4, then translocates into the nucleus to bind with target genes to initiate transcription [8,17,24]. Simultaneously, TGF-β1 induces the expression of a Smad ubiquitin regulatory factor (Smurf) that degrades Smad7, an inhibitory Smad that competes with Smad3 and Smad2 for binding to TβRI to reinforce TGF-β1/Smad signaling [25].

## 3. TGF-β1 Signaling in Kidney Diseases

TGF-β1 is primarily involved in a dynamic pathophysiological process that leads to renal fibrosis. It is significantly upregulated in the injured kidney as a primary step in tissue scarring [8,26,27] and the progressive forms of kidney disease [28]. The latest studies have revealed the diverse roles of TGF-β1, for instance as a major inducer of macrophage polarization [23], myofibroblast differentiation, and accumulation in the fibrotic kidney, which is primarily reduced by conditional deletion of TGFβ receptor 2 (Tgfbr2) [29,30,31].

TGF-β1 acts on both residential kidney cells (e.g., renal tubule epithelial cells, mesangial cells, and podocytes) and infiltrated immune cells (macrophages and T cells) to promote fibrotic progression via inducing apoptosis, ECM protein synthesis and secretion, and trans-differentiation for de novo myofibroblast generation, a specialized cell type that actively secretes collagens for ECM deposition [30,32,33]. For instance, TGF-β1 induces podocytopenia via apoptosis of podocytes [34], resulting in the progression of glomerulosclerosis [35]. Furthermore, TGF-β1 directly triggers ECM production from renal fibroblasts, mesangial cells, and podocytes [36,37,38] and further generates collagens producing myofibroblasts by trans-differentiating tubular epithelial cells, endothelial cell, and bone marrow-derived macrophages via epithelial-mesenchymal transition (EMT) [39], endothelial-mesenchymal transition (EndoMT) [40], and macrophage–myofibroblast transition (MMT)[30,41] in the fibrotic kidney. TGF-β1 in the diseased kidney activated these processes to accelerate fibrotic progression dramatically.

Smad3 is the canonical downstream of TGF-β1 signaling, serving as a key mediator of kidney fibrosis by promoting myofibroblast accumulation and fibrogenic molecule production in multiple experimental renal diseases, which is dramatically suppressed by Smad3 deletion [42,43,44,45]. Increasing evidence suggests that macrophages are a key player in the Smad3-dependent fibrogenic progression, particularly via the direct mechanism of MMT [46,47]. In a chimeric study with Smad3^−/−^ and Smad3^+/+^ GFP^+^ bone marrow transplanted into irradiated mice with unilateral ureteral obstruction (UUO), Smad3-deleted macrophage (Smad3^−/−^ GFP^+^ F4/80^+^) failed to undergo MMT to generate myofibroblasts (GFP^+^ α-SMA^+^) for collagen-I deposition in the fibrotic kidney, which is in contrast to the profound MMT activity of Smad3 wildtype macrophage (Smad3^+/+^ GFP^+^ F4/80^+^) [48]. These findings demonstrate that Smad3 is the key regulator of MMT [48]. However, targeting Smad3 may cause dysregulation of the immune system, contributing to the development of autoimmune disease [49]. Thus, Smad3 direct downstream targets responsible for pathogenic processes including MMT were identified for developing an antifibrotic strategy with minimal side effects. Single-cell RNA seq resolved the cell-cell transcriptome of MMT, revealing proto-oncogene tyrosine-protein kinase Src- and neural transcription factor Pou4f1-centric regulatory gene networks driving MMT in the injured kidney in vivo and TGF-β1-induced bone marrow-derived macrophages in vitro [30,41]. Further molecular study reveals that Src and Pou4f1 are the direct targets of Smad3, where Src inhibition and macrophage-specific Pou4f1 silencing mimicked the protective effect of Smad3^−/−^ in MMT suppression and associated myofibroblast generation and collagen-I production, representing a precision strategy for targeting MMT [30,41]. Owing to lncRNA’s temporal and spatial specificity, Smad3-dependent LncRNA-regulating MMT may be identified to further enhance the precision of targeted MMT antifibrotic therapy.

### 3.1. TGF-β1-Associated lncRNAs in Kidney Diseases

#### 3.1.1. lncRNAs in TGF-β1 Induced EMT

**Figure 1:**
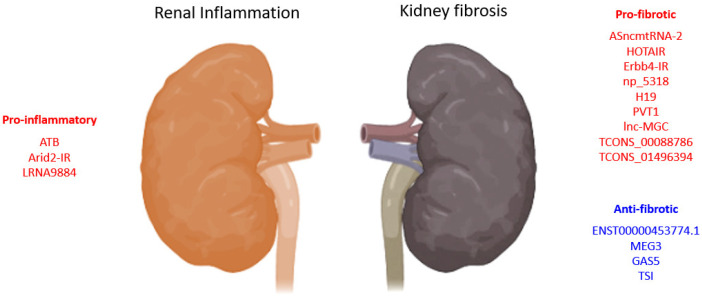
The roles of TGF-β1-dependent lncRNAs in renal disease progression. In the development of chronic renal disease, TGF-β regulates renal inflammation and fibrosis via inducing lncRNAs, which are potential therapeutic targets against CKD development.

Studies in recent decades revealed that lncRNA is one of the pathogenic downstream regulators of TGF-β1 signaling in inflammatory diseases [4,18] (Figure 1). The majority of lncRNAs exert their biological effects by altering transcriptional or posttranscriptional processes such as transcription factor recruitment, RNA maturation, protein synthesis, and transport. These lncRNAs are also capable of changing chromatin structure via polycomb repressive complex 2 (PRC2) and repressing miRNAs via complementary binding (sponging) [50,51]. For instance, lncRNAs and protein-coding genes were induced via similar mechanisms that shared histone-modification profiles and exon/intron architecture, but lncRNAs are predominantly localized in the nucleus and expressed at a lower level, although with higher tissue specificity compared with coding genes [52]. Most novel lncRNAs were discovered in primates with high-throughput RNA sequencing, revealing disease-associated TGF-β1-dependent lncRNAs that were significantly upregulated in experimental disease conditions in vivo and in vitro [53] (Table 1). EMT is a crucial pathogenic process in kidney fibrosis. Cell-cell connections between tubular epithelial cells were progressively lost and ECM molecules were actively produced by EMT-derived cells to transform nephrons into functionless scar tissue [39]. Numerous studies suggested that TGF-β1-dependent lncRNAs regulate EMT. LncRNAs have been proposed to modulate gene expression indirectly as competing endogenous RNAs (ceRNAs), where they compete with a network of mRNAs and circular RNAs (circRNAs) to bind to microRNAs [54]. LncRNAs acting as ceRNAs may represent significant modulation of the canonical TGF-β pathway. PVT1 (plasmacytoma variant translocation 1) is the first lncRNA identified to be associated with diabetic nephropathy, where single-nucleotide polymorphisms (SNPs) significantly associated with end-stage renal disease (ESRD) of type 2 diabetes were located in PVT1 [55]. Further study reveals that high glucose levels induced PVT1 to stimulate TGF-β1, PAI-1, and FN1 expression, which is further amplified by PVT1-derived miR-1207-5p to accelerate ECM accumulation in the diseased glomeruli of diabetic nephropathy (DN) [56]. lncRNA-MGC (megacluster) is a host of 40 miRNAs upregulated in the TGF-β1-treated mesangial cells in vitro and the glomeruli of diabetic mice in vivo via transcription factor CHOP [57]. Inhibiting MGC by antisense oligos GapmeRs in the diabetic kidneys of streptozotocin-injected mice effectively suppressed a cluster of miRNAs and profibrotic gene expression (Col1a2, Col4a1), contributing to the dramatic reduction of PAS-positive areas, glomerular basement membrane (GBM) thickness, and terminal deoxynucleotidyl transferase dUTP nick end labeling (TUNEL)-positive cells [57]. These results suggest a role of miRNAs in the regulation of MGC-driven DN progression.

In one study, lncRNA-HOTAIR (HOX transcript antisense RNA) was upregulated in UUO kidneys in vivo and TGF-β1-stimulated HK-2 cells in vitro, associated with the Notch signaling (JAG1, Notch1, NICD) and switching of EMT-related proteins, for example, alpha-smooth muscle actin (α-SMA), fibronectin (FN), and E-cadherin [58]. Further analysis shows that HOTAIR sponged miR-124 via a conserved binding site, thus preventing Notch signaling suppression, demonstrated by how the silencing of HOTAIR prevented TGF-β1-induced Notch signaling and EMT, but both were restored by further application of miR-124 inhibitor into the HOTAIR-silenced group [58]. Separately, lncRNA-MEG3 (maternally expressed gene 3) is a protective lncRNA downregulated in TGF-β1-stimulated HK-2 cells, where TGF-β1 suppressed miR-185 to induce CpGs methylation of MEG3 promoter via DNA methyltransferases 1 (DNMT1) [59]. Overexpression of MEG3 largely suppressed TGF-β1-induced apoptosis and EMT [59]. TCONS_00088786 was another pro-fibrotic lncRNA upregulated in the UUO kidney, where TCONS_00088786 silencing effectively suppressed collagen I and III, and profibrotic miR-132 expression [60].

**Table 1:** The TGF-β-associated lncRNAs in renal diseases.

LncRNA	Biological Process	Model	Species	Mechanism	Year	Ref.
lnc453774.1	anti-fibrosis	HK-2 cells	Human	associated with ceRNAs targeting FBN1, IGF1R, KLF7 PPI networks	2021	[61]
ATB	pro-inflammation	HK-2 cells	Human	promotes apoptosis, senescence, inflammatory cytokines (TNF-α, IL-1β, and IL-6), and adhesion molecules (VCAM-1 and sE-selectin) expression	2020	[62]
HOTAIR	pro-fibrosis	UUO, TECs-HK-2	Human	promotes EMT via Notch1 and miR-124	2019	[58]
ENST00000453774.1	anti-fibrosis	Renal biopsy, UUO, TECs-HK-2	Human	promotes autophagy (Atg5/7) and Nrf2-driven HO-1 expression and suppresses ECM synthesis (Fn, Col-I)	2019	[63]
MEG3	anti-fibrosis	HK-2 cells	Human	suppresses EMT of HK2 cells and is regulated by miR-185/DNMT1/MEG3 pathway	2019	[59]
TCONS_00088786	pro-fibrosis	UUO, NRK52E cells	Rat	promotes collagen I, III, and miR-132 expression	2018	[60]
	pro-fibrosis	RNA-seq of rat UUO, NRK52E cells	Rat	promotes Col1a1 and Col3a1 expression	2017	[64]
TCONS_01496394				promotes Ctgf and Fn1 expression		
ASncmtRNA-2	pro-fibrosis	HRMC, DN	Human, mouse	promotes TGF-β and Fn1 expression	2017	[65]
lnc-MGC	pro-fibrosis	STZ-DN, MMC, MCs	Human, mouse	host of miRNA mega-clusters regulating profibrotic genes expression	2016	[57]
PVT1	pro-fibrotic	MC, RPTEC, podocytes	Human	PVT1-derived miR-1207-5p-induced TGF-β1, PAI-1, and FN1	2013	[56]
	pro-fibrotic	ESRD-T2D GWAS	Human	23 SNPs associated with ESRD	2007	[55]

ceRNAs: competing endogenous RNAs, FBN1: fibrillin-1, IGF1R: insulin-like growth factor 1 receptor, KLF7: Kruppel-like factor 7, PPI: protein-protein interaction, ATB: activated by transforming growth factor-β, TNF-α: tumor necrosis factor alpha, IL: interleukin, VCAM-1: vascular cell adhesion molecule 1, HOTAIR: HOX transcript antisense RNA, UUO: unilateral ureteral obstruction, EMT: epithelial-mesenchymal transition, ECM: extracellular matrix, Fn: fibronectin, Col: collagen, MEG3: maternally expressed gene 3, Ctgf: connective tissue growth factor, ASncmtRNA-2: antisense mitochondrial non-coding RNA-2, HRMC: human renal mesangial cell, DN: diabetic nephropathy, MGC: megacluster, STZ: streptozotocin, MMC: mouse mesangial cell, MCs: mesangial cells, PVT1: plasmacytoma variant translocation 1, RPTEC: human renal proximal tubule epithelial cells, PAI-1: plasminogen activator inhibitor 1, ESRD: end-stage renal disease, T2D: type 2 diabetes, GWAS: genome-wide association studies, SNPs: single nucleotide polymorphisms.

#### 3.1.2. lncRNAs Associated with Reactive Oxygen Species

Reactive oxygen species (ROS) are another mechanism leading to fibrotic progression. ROS that accumulate during acute kidney injury cause damage to tubular epithelial cells and the release of pro-inflammatory cytokines, and renal inflammation and fibrosis develop if oxidative stress persists [66,67,68]. Antioxidative and autophagy systems were cellular defense mechanisms against oxidative stress by removing ROS and damaged organelles to limit oxidative damage [69]. LncRNA-ATB was found to be highly expressed in TGF-β1-induced HK-2 cells to promote inflammatory cytokines (TNF-α, IL-1β, and IL-6), adhesion molecules (VCAM-1 and sE-selectin), and pro-senescence factor (p53/p21/p16) expression [62]. In contrast, protective lncRNA 74.1 (ENST00000453774.1) was identified from downregulated differentially expressed genes (DEGs) of TGF-β1-treated HK-2 cells, where it is largely suppressed in the fibrotic tissues compared with the normal control in human renal biopsy [63]. Overexpression of LncRNA 74.1 activates Nrf2/HO-1 antioxidant and Atg5/Atg7/LC3 autophagy pathways in TGF-β1-treated cells, contributing to the protective effect of LncRNA 74.1 overexpression against UUO-induced fibrosis in vivo [63]. ROS are also involved in the pathogenesis of DN, causing mesangial matrix expansion and thickening of the glomerular basement membrane. The lncRNA-ASncmtRNA-2 (antisense mitochondrial non-coding RNA-2) was upregulated in DN of Lepr^−/−^(db/db) mice in vivo and high glucose-stimulated mesangial cells in vitro, promoting TGF-β1 and fibronectin expression in a ROS-dependent mechanism, where shRNA-mediated ASncmtRNA-2-silencing and ROS inhibition by NG-nitro-L-Arginine methylester (L-NAME) effectively suppressed DN and high glucose-induced TGF-β1 and fibronectin expression [65]. Yuan et al. recently identified lnc453774.1(ENST00000453774.1) in TGF-β1-stimulated human kidney epithelial cells, revealing a lnc453774.1-centric fibrotic gene network, interacting with 14 competing endogenous miRNAs to control 8 key functional genes for autophagy, oxidative stress, and cell adhesion (FBN1, IGF1R, KLF7, etc.), suggesting a key regulatory role of lnc453774.1 [61].

### 3.2. Smad3-Dependent lncRNAs in Kidney Diseases

Smad3 plays an important role in TGF-β-driven renal inflammation and fibrosis, but potential side effects in vital organs limit its therapeutic application [70]. Therefore, Lan’s group further identify several Smad3 downstream profibrotic lncRNAs as therapeutic targets against renal fibrosis via RNA sequencing [71] (Table 2). Smad3-WT-specific upregulated lncRNAs were extracted from the Smad3-dependent transcriptomes of both UUO and anti-GBM kidneys, eventually revealing 21 potential fibrogenic lncRNAs suppressed by Smad3 deletion [71]. The lncRNAs GAS5 [72], LRNA9884 [73], and lnc-TSI [74] are Smad3 direct targets, regulated by its direct binding to the regulatory sequence of lncRNAs as detected by ChIP-PCR assay, and its influence on the expression levels of lncRNAs was further confirmed by luciferase reporter assay. Smad3 transcriptionally regulates LRNA9884 in the advanced glycation end product-stimulated embryonic fibroblasts (MEFs) and kidney of diabetic mice (db/db) via direct binding on LRNA9884 promoter, promoting MCP-1-mediated renal inflammation via direct binding to its promoter [73]. Moreover, LRNA9884 is also involved in the pathogenesis of acute kidney injury (AKI), which is highly expressed in the tubular epithelial cells of AKI kidneys, promoting IL-1β-induced inflammatory cytokine production (MCP-1, TNF-α, and IL-6) via transcriptional regulation of macrophage migration inhibitory factor (MIF) to trigger MIF/NF-κB pathway [75]. Therefore, LRNA9884 inhibition might be a potential therapy for DN and AKI.

LncRNA Ptprd-IR (np_4334, intron of protein tyrosine phosphatase receptor delta), is one of the 21 Smad3-WT-specific upregulated lncRNAs under fibrotic conditions [71]. Smad3 transcriptionally regulates Ptprd-IR expression in TGF-β1-stimulated mouse renal tubular epithelial cells (mTECs) and UUO kidneys via a conserved Smad3 binding site on Ptprd-IR’s promoter [76]. Interestingly, Ptprd-IR promotes TGF-β1- and IL-1β-mediated activation of the NF-κB pathway, resulting in pro-inflammatory cytokine production and renal inflammation in UUO kidneys in vivo and mTEC in vitro, while it has no effect on TGF-β1-induced renal fibrosis [76].

LncRNA Erbb4-IR (intron of Erb-B2 receptor tyrosine kinase 4) is expressed in diabetic kidneys of db/db mice, and AGEs stimulated MEFs via a Smad3-dependent mechanism [71,77]. Erbb4-IR promotes fibrotic progression of a diabetic kidney in vivo, and Col-I/IV expression in AGE-stimulated mouse mesangial cells and tubular epithelial cells in vitro, via sponging renal protective miR-29b through direct binding to its 3’ untranslated region (UTR) [80]. In a UUO kidney and its in vitro model, Smad3 regulates Erbb4-IR to promote Col-I and α-SMA expression via suppressing Smad7, a suppressor of TGF-β/Smad3 signaling by direct interaction with the 3’ UTR of Smad7. Therefore, renal Erbb4-IR silencing effectively restored Smad7 expression against UUO-induced fibrotic progression [80]. This also applied to the pathogenesis of ischemia-reperfusion-induced AKI, where TGF-β/Smad3 signaling was further amplified by Erbb4-IR [78].

Smad3 transcriptionally regulates lncRNA Arid2-IR (np_28496 [81]) expression in UUO kidneys and TGF-β-induced mTEC via direct binding on the promoter region of Arid2-IR. Interestingly, Arid2-IR promotes inflammatory response instead of fibrosis, where NF-κB-driven inflammatory cytokine expression was largely suppressed by Arid2-IR silencing [81], confirming Arid2-IR as a Smad3-associated lncRNA that promotes renal inflammation via crosstalk with the NF-κB pathway [81]. Moreover, LncRNA-TSI directly binds to the MH2 domain of Smad3 to prevent its phosphorylation by TGF-β1 receptor I, thus suppressing Smad3-dependent profibrotic signaling [74]. In addition, J. Sun et al. also revealed 24 upregulated lncRNA candidates by transcriptome analysis of UUO- and Sham-operated renal tissues in which 2 lncRNAs, TCONS_00088786 and TCONS_01496394, contain 4 conserved Smad3 binding motifs and are detected in TGF-β1-stimulated renal tubular epithelial NRK-52E cells [64]. Their pathogenic role in renal fibrosis was confirmed by gene silencing, where TGF-β–induced expression of profibrotic molecules Col1a1 and Col3a1 was regulated by TCONS_00088786, while Ctgf and Fn1 were controlled by TCONS_01496394 [64]. In addition, Smad3-dependent antifibrotic lncRNA GAS5 (growth arrest-specific 5) was largely suppressed in a UUO kidney and TGF-β1-induced mTEC, contributing to the Col-I and Fn expression. Further mechanistic study reveals that GAS5 interacted with miR-142-5p, which binds to the 3′UTR of Smad3 to suppress TGF-β1-induced apoptosis and Col-I/Fn expression of mTEC [72].

## 4. TGF-β1 Signaling in Tumor Progression

**Figure 2:**
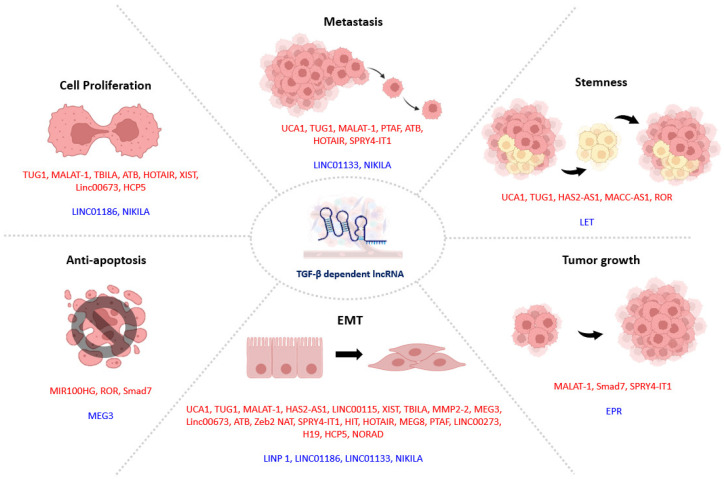
Diverse mechanisms of TGF-β-dependent lncRNA in cancer progression. Pleiotropic TGF-β regulates tumor progression via inducing pathogenic (red) and protective (blue), which modulate cancer cell activities from the early to the advanced stages. Notably, stemness of cancer cells largely contributes to treatment resistance and recurrence.

The role of TGF-β1 is also pleotropic in the tumor microenvironment (TME), simultaneously regulating both pro- and anticancer processes during tumor progression. TGF-β1 signaling activation has been associated with metastasis and poorer prognosis due to the induction of EMT and drug resistance [82,83], but disruption of TGF-β1 signaling also leads to poor prognosis and accelerated tumor progression as TGF-β1-induced cancer cell apoptosis was relieved [84]. EMT is induced in epithelial tumor cells with prolonged exposure to TGF-β1, phenotypic changes including the loss of cell-cell adhesion between epithelial cancer cells to allow their migration and invasion, and the acquisition of protumoral cancer-associated fibroblast phenotype [22,85]. Thus, EMT is a critical step in cancer metastasis and a critical contributor to patients’ poor prognosis. At the molecular level, EMT is featured by the downregulation of E-cadherin and upregulation of the EMT markers N-cadherin, Vimentin, α-SMA, and EMT-associated transcription factor SNAI1/2 and ZEB1 [86]. Moreover, TGF-β1 promotes drug resistance by upregulating slow-cycling cancer stem cells (CSCs) that escape from chemotherapy and contribute to the recurrence [83,87]. Interestingly, TGF-β also induces the apoptosis of cancer cells by inhibiting the cell cycle via expression of cyclin-dependent kinases including p15^Ink4b^, p21^Cip1^, and p57^Kip2^ and pro-apoptotic factor Bim [88,89]. Therefore, targeting the pathogenic downstream effectors of TGF-β1 would selectively suppress its pro-tumoral effects without interfering with the anticancer effect of TGF-β1; LncRNA with high tissue and disease specificity represents an ideal TGF-β1-targeting strategy for anticancer therapy (Figure 2).

### 4.1. TGF-β1-Dependent lncRNAs in Tumor Progression

#### 4.1.1. lncRNAs in TGF-β1 Induced EMT

Studies in the past decade have identified a number of TGF-β1-induced lncRNAs in different types of cancer (Table 3). Most TGF-β1-induced lncRNAs were found to be involved in the regulation of EMT, drug resistance, apoptosis, and the proliferation of cancer cells. TGF-β1 induces the LncRNA MALAT-1 (metastasis-associated lung adenocarcinoma transcript 1) in several types of cancer to promote EMT via different downstream mechanisms [90,91,92,93,94,95,96,97]. In metastatic bladder cancer, MALAT1 directly interacts with suz12 (a component of histone-modifying complex-PRC2) to regulate the expression of EMT-associated genes (E-/N-cadherin, fibronectin, MMP9), contributing to the migration and metastasis of cancer cells in vitro and in vivo [95]. One study of aggressive renal cell carcinoma further reveals that MALAT1 enhances the levels of Ezh2 and H3K27me3 on E-cadherin promoter region, epigenetically suppressing E-cadherin to promote EMT [93]. In addition to EMT, MALAT1 promotes metastasis by enhancing angiogenic hepatocellular carcinoma cells; VEGF-A expression and associated angiogenesis in vivo were regulated by MALAT1 in a miR-140-dependent manner [90]. Moreover, MALAT1 also controls the bioavailability of TGF-β1 in the extracellular space by activating the transcription of latent TGF-β binding protein 3 (LTBP3) via recruiting Sp1 to its promoter [96].

The lncRNA HOTAIR interacts with PRC2 complex to alter the chromatin state for the metastatic phenotype of breast cancer cells [135]. HOTAIR promotes PRC2 occupancy on the promoters of 854 genes detected by genome-wide promoter array assay on ChIP enriched with EZH2, H3K27me3, and SUZ12 target genes in HOTAIR-overexpressing cells [135]. Among the PRC2-associated genes, 35 genes are associated with the stemness of cancer cells, contributing to the TGF-β1-induced upregulation of CD133^+^/CD44^+^ CSC populations [134]. In renal carcinoma, HOTAIR regulates the proliferation and invasiveness of cancer cells by promoting H3K27me3 in cell cycle-related genes’ promoters (p53, p21, and p16) via EZH2 and by sponging miR-141 to regulate ZEB1 expression [131,132]. Interestingly, lncRNA MEG3 brings the GA-rich distal regulatory element and PRC2 into close proximity via two interacting sequences encoded to target the GA-rich region and the PRC2 complex, thus modulating the transcription of the TGF-β pathway genes [119]. Further analysis of lung cancer cell lines reveals that MEG3 epigenetically suppressed E-cadherin and miRNA-200s via recruitment of JARID2 and EZH2 and associated histone H3 methylation [117]. The lncRNA ATB promotes the EMT of several types of cancer cells [4,124,126,127] via sponging miR-200s, an EMT suppressor inhibiting ZEB1/2 expression via direct binding to their 3′UTR, which is detected between ATB and miR-200a/b/c in an RNA immunoprecipitation (RIP) experiment [4,138,139]. The ATB/miR-200s/ZEB1/2 axis was demonstrated in the EMT of hepatocellular carcinoma and gastric and breast cancer cells [4,125,126]. Moreover, in HCC cells, ATB also interacts with the IL-11 transcript to extend its half-life, facilitating the synthesis and secretion of IL-11 into the supernatant as the autocrine signal for STAT3 activation [4]. This IL-11/STAT3 axis is essential for the metastatic colonization of the lung and liver [4].

LncRNAs regulate miRNAs targeting key activators of EMT to drive the phenotyping changes in cancer cells. ZEB1/2 are transcriptional repressors of E-cadherin, which is essential for the initiation of EMT [139,140]. ZEB1 is regulated by LINC00673 and LINC00273 via sponging miR-150-5p and miR-200a-3p, respectively, to reduce the binding of miRNAs on the 3′UTR of ZEB1 [103,120]. Meanwhile, ZEB2 and Notch-1 are regulated by XIST via miR-367/miR-141 and miR-137, respectively, in NSCLC cells, contributing to increased pulmonary metastasis nodules in vivo and cell migration in vitro [113,141]. Interestingly, the transcription of ZEB2 is also regulated by its natural antisense transcript ZEB2 NAT, which overlaps the splicing site at 5′UTR of ZEB2 as a potential internal ribosome entry site (IRES) sequence to promote ZEB2 translation, accounting for the EMT of colon adenocarcinomas and urinary bladder cancer cells [128,129]. In addition to NAT, the long non-coding transcript of MMP2 (LncRNA-MMP2-2) is also induced by TGF-β1 and contained in exosomes to promote host gene MMP2 expression for enhancing cancer cell migratory ability [114]. Likewise, the lncRNA miR-155 host gene (MIR155HG) promotes EMT via derived miR-155-5p, which targets the 3’ UTR of SOX10 to regulate its transcription. Moreover, zinc finger protein SNAI1/2 is a powerful initiator of EMT induced by TGF-β1-associated lncRNAs for suppressing E-cadherin expression [107,108,129]. In ovarian cancer cells, SNAI2 expression is upregulated by PTAF via sponging miR-25, which suppresses SNAI2 expression via direct binding to its 3′UTR [115]. In lung and pancreatic cancer cells, SNAI1/2 is upregulated by MEG8 via suppressing miR-34a and miR-203 through histone H3 methylation of their regulatory regions [116]. SNAI1 expression is also induced in esophageal squamous carcinoma cells by lncRNA SPRY4-IT1, driving the EMT of thyroid cancer cells and contributing to metastasis and poor patient prognosis [136,137]. Another EMT-regulating transcription factor Twist is upregulated in colorectal and pancreatic cancer cells by TGF-β1-induced lncRNA TUG1, suppressing E-cadherin expression to promote the migration capacities of these cancer cells [104,105]. Serine/arginine-rich splicing factor 6 (SRSF6) is highly expressed in the cancer tissues of patients with metastatic tumors and is induced by TGF-β1 via suppressing SRSF6 repressing lncRNA LINC01133 [123].

#### 4.1.2. lncRNAs Associated with TGF-β1-Induced Drug Resistance

Cancer stem cells (CSCs) are a subpopulation with self-renewal and high tumorigenic capacity that contribute to TGF-β1-induced drug resistance [83]. LncRNA UCA1 promotes the stemness and proliferation of cancer cells for escaping from anticancer treatment, where stemness regulators Nanog, ALDH1, and HXK2 expression were regulated by the UCA1/miR-1, miR-203/Slug axis [99,100]. UCA1 also promotes PD-L1 expression in anaplastic thyroid carcinoma cells to suppress CD8+ T-cell-mediated cell cytotoxicity via sponging miR-148a to prevent its suppression on PD-L1 expression [98]. miRNA-145 plays a suppressive role in CSC formation, which is inhibited by LET and MACC1-AS1 against the gemcitabine resistance of bladder cancer cells and the 5-FU and oxaliplatin resistance of gastric cancer cells, respectively [110,122,142]. In breast cancer cells, the lncRNA Has2as mediates TGF-β-induced stemness via regulating a panel of stemness-associated transcription factors (Pou5f1, Sox2, Nanog, Zfp42). In glioma cells, lncRNA LINC00115 promotes the sphere formation of stem-like cancer cells in vitro and tumor growth in vivo via sponging miR-200s to reduce their binding on the 5′UTR of ZNF596, a downstream stemness regulator of glioma cells [109]. The lncRNAs CASC11 and HAND2-AS1 were upregulated in small cell lung cancer (SCLC) and non-small cell lung cancer cells (NSCLC), respectively, enhancing TGF-β1 expression to promote a CDD133+ CSC population of SCLC cells [106,107]. In hepatocellular carcinoma cells, TGF-β1 induced the enrichment of lincRNA-ROR (linc-ROR) in extracellular vesicles, promoting the formation of spheroid CD133^+^ stem-like cells against sorafenib-, doxorubicin-, and camptothecin-induced cancer cell death [130]. Another lncRNA, H19, promotes CSCs and their formation of spheroid HCC cells, which is regulated by the noncanonical TGF-β/TGFBR2/SOX2 and PI3K/AKT/miR-675 axis [101,102].

### 4.2. Smad3-Associated lncRNAs in Tumor Progression

Smad3 is a pathogenic mediator of TGF-β1 signaling in tumor progression [22,143,144,145]. lncRNAs are associated with the pathogenic action of Smad3 as shown in Table 4. The lncRNA NORAD (LINC00657) directly interacts with importin β1 to facilitate the nuclear localization of Smad3 and its associated complex for the transcription of EMT-related genes [146]. The lncRNA EPR (epithelial cell program regulator) promotes epithelial trait preservation and suppresses cancer cell proliferation via cyclin-dependent kinase inhibitor 1A (Cdkn1a) expression, which is regulated by EPR through enhancing the SMAD3-Cdkn1a promoter binding and preventing KHSRP- (KH-type splicing regulatory protein) mediated destabilization of Cdkn1a mRNA [147]. The LncRNAs TBILA [148], NKILA [141,149], and HCP5 [150] were induced by TGF-β1 and transcriptionally regulated by Smad3 via physical binding to its promoter; these lncRNAs then cross-talked with other signaling pathways including S100A7/JAB1 [148], NF-κB/Snail [149], NF-κB/MMP14 [141], and miR-203/SNAI [150] to further activate EMT-related gene programs to complete changes into metastatic phenotypes. Interestingly, Smad3 regulates the transcription of the lncRNA MIR100HG and the derived miRNA miR-100/-125b, which form miR-100- and miR-125b-centric regulatory networks of TGF-β response genes in PDAC cells to control the pathways associated with p53/apoptosis/tight junction and cell-cycle checkpoint induced by TGF-β [151]. Smad7 inhibits the phosphorylation of Smad3 to prevent its transcription activity [152]. The antisense strand of Smad7 upstream was transcripted into lncRNA-Smad7, which specifically suppresses TGF-β-induced apoptosis of cancer cells, but TGF-β-induced EMT was not affected [153]. Smad4 forms a complex with phosphorylated Smad3 for nuclear localization and transcription. LncRNA LINP1 is suppressed by TGF-β in a Smad4-dependent manner, responsible for epithelial phenotype maintenance via suppressing EMT [154].

## 5. Therapeutic Strategies Targeting lncRNAs

Due to the dual roles of TGF-β1 in physiological and pathological contexts, targeting TGF-β1 is not an optimal therapeutic strategy as there is evidence that TGF-β1 deficiency might impair host immunity and cause autoimmune diseases [26]. Although many studies have demonstrated that blocking TGF-β1 protects against progressive renal fibrosis and cancer, others have also highlighted the potential consequences of TGF-β1 inhibition, for instance lethal inflammation observed in TGF-β1-deficient mice at 3 weeks of age [155]. Currently, TGF-β1 inhibitors have not been approved for cancer or fibrosis therapy due to reported cytotoxicity in recent clinical trials. A case in point is fresolimumab, a human anti-TGF-β1 monoclonal antibody (mAb), was found to have no significant effect on proteinuria, eGFR, or serum creatinine in focal and segmental glomerulosclerosis (FSGS) [156]. Furthermore, side effects including pustular rash, herpes zoster, bleeding, skin lesions, and cancer have also been observed after anti-TGF-β therapies. Of note, fresolimumab was found to be involved in the development of cutaneous lesions, and results showed that keratoacanthomas were the most common cutaneous neoplasms observed as adverse events in therapies targeting TGF-β [157]. Therefore, targeting the downstream effector of TGF-β may represent a therapeutic strategy specifically against the pathogenic effects of TGF-β without major disturbances to the immune system. Much insight has been gained into how lncRNAs regulate processes such as fibrosis, tumorigenesis, and ECM accumulation, where they can act via binding to Smad proteins, serving as miRNA sponges or interacting with other signaling pathways. These lncRNAs can be used for diagnosis and targeted when their pathogenic mechanisms are elucidated. Findings from preclinical studies have shown the potential of targeting TGF-β-associated lncRNAs for treating kidney diseases and cancers.

Smad3-dependent lncRNAs with therapeutic potential in renal diseases have been identified in previous studies. Inhibition of Erbb4-IR alleviated renal fibrosis in fibrotic UUO and DN models [79,80,158]. Inhibition of Arid2, LncRNA_5318, and LRNA9884 also suppressed renal inflammation in UUO and diabetic models [73,75,81,159]. For antineoplastic therapy, the modulation of lncRNAs not only regulates the TME but also participates in combination with first-line therapy. Gemcitabine and cisplatin are standard therapy for advanced/metastatic carcinoma, where LINC01714 dramatically enhanced the gemcitabine sensitivity of cholangiocarcinoma cells [160]. LncRNA can be targeted by antisense-based strategies or by shRNAs, consisting of siRNAs and modified antisense oligonucleotides (ASOs) [161]. The ASO-based technologies including novel chemical modifications were optimized with multiple preclinical trials, and the efficiency of cellular uptake and the expression levels of targeted ncRNAs have largely improved [162]. The inhibition efficiency and toxicity were major concerns of directly administering lncRNA-targeting agents via tissue or tail vein injection, where toxicity is observed in a dose-dependent manner, i.e., off-target effects through nonspecific binding to similar nucleotide sequences [163,164,165]. However, repeated high dosages of siRNAs and gapmers are required for effective lncRNA inhibition in vivo. Therefore, post-delivery monitoring and optimizing effective concentration of lncRNAs therapeutics are critical for translational application. Novel noninvasive ultrasound microbubble-assisted (USMB) delivery largely reduced the concentrations of lncRNA-targeting agents in nontargeted tissue [166], contributing to the safety and effectiveness in preclinical studies [73,80,167]. Thus, USMB represents a realistic approach to translating lncRNA-targeted therapeutics with added value in postdelivery monitoring and assessment with its imaging function. Moreover, among the FDA- (Food and Drug Administration) and EMA- (European Medicines Agency) approved ASO-based therapies targeting mRNA expression in the liver, most were administered subcutaneously (mipomersen, inotersen, givosiran, volanesorsen, inclisiran, and lumasiran) [5], suggesting the potential for developing subcutaneously delivered lncRNA-targeted ASOs for kidney fibrosis and cancer.

Collectively, lncRNA-targeted therapy might represent an effective strategy for fibrosis and cancer due to its superior tissue and disease specificity. However, the translation of lncRNA-targeted therapy was limited by the dose-dependent toxicities associated with the delivery of lncRNA therapeutics and the lack of conservation among species; that is, human lncRNAs may lack mouse homologs for preclinical study, and mouse lncRNAs may lack human homologs for therapeutic development [168]. Therefore, an effective approach in identifying homologues among species or using humanized mouse models may facilitate the translation of experimental findings into preclinical settings for lncRNA-based therapeutics development [169]. In addition, TGF-β-dependent lncRNAs are disease specific, and in circulation, these lncRNAs are biomarkers of associated diseases. The FDA approved the use of the lncRNA PCA3 in urine as a biomarker for detecting prostate cancer with high sensitivity [170]. As the circulating levels of the TGF-β-associated lncRNAs UCA1 [171], H19 [172], and MALAT1 [173] are associated with disease progression, the potential of these lncRNAs for use as biomarkers for diagnosis could be further explored.

## 6. Conclusions

The translational development of therapeutics targeting the TGF-β1 signaling pathway has been largely hindered by its key regulatory roles in multiple physiological processes. In recent decades, the dissection of TGF-β1 signaling pathways has revealed numerous precise therapeutic targets, including lncRNAs for inflammatory diseases. Emerging evidence shows that lncRNAs are specific pathogenic mediators of TGF-β1, regulating a particular function of TGF-β1 during inflammatory disease progression that can be targeted to develop effective gene-based therapies. With the advancement of RNA sequencing at single-cell resolution and bioinformatic analysis, a more in-depth regulatory mechanism of lncRNAs in inflammatory diseases will be discovered. Disease- and cell-type-specific lncRNAs will be identified for the development of precision therapies against tissue inflammation and cancers.

## Figures and Tables

**Table 2:** The Smad3-associated lncRNAs in renal diseases.

LncRNA	Biological Process	Model	Species	Mechanism	Year	Ref.
GAS5	anti-fibrosis	Smad3-WT/KO UUO, mTECs, MEFs	Mouse	suppresses TGF-β1-induced Col-I/Fn expression and apoptosis, promotes miR-142-5p expression	2021	[72]
LRNA9884	pro-inflammation	Cisplatin-AKI, mTECs	Mouse	promotes IL-1β-induced p-p65,TNF-α, MCP-1, and IL-6, binds directly to MIF promoter	2020	[75]
		Smad3-WT/KO-DN, mTECs, SV40 MES 13	Mouse	Smad3 dependently induced, suppresses IL-1β, TNF-α, and MCP-1, binds directly to the promoter of MCP-1	2019	[73]
Ptprd-IR (np_4334)	pro-inflammation	mTECs, HEK293T, UUO mice	Human, mouse	Smad3 direct target; promotes inflammatory response and macrophage and T-cell infiltration	2020	[76]
Erbb4-IR (np_5318)	pro-fibrotic	Smad3-WT/KO-DN, TECs, MCs	Mouse	Smad3 deletion suppressed Erbb4-IR and restored miR-29b expression	2020	[77]
		AKI, PCS-400-012 cells	Human, mouse	promotes I/R-induced renal cell death, further enhances TGF-β1/Smad3 signaling	2020	[78]
		UUO, TEC, MEF	Mouse	suppresses Smad7 via promoter binding, enhances Smad3-driven Col-I α-SMA expression	2018	[79]
		Smad3-WT/KO-DN, TECs, MCs, MEF	Mouse	enhances Smad3-driven Col-I/IV expression, suppress protective miR-29b via 3’UTR binding	2018	[80]
TSI	anti-fibrosis	UUO, HK2, TECs, MC, HL-7702, LX-2, IMR-90, 16HBE, HKC8 cells	Human, mouse	inhibits Smad3 by direct binding to MH2 domain	2018	[74]
Arid2-IR	pro-inflammation	UUO, TEC	Mouse	Smad3 direct target; promote fibrotic and inflammatory response, macrophage and T-cell infiltration	2015	[81]
RNA-seq	pro-fibrotic	UUO /anti-GBM GN of Smad3-WT/KO mice	Mouse	21 TGF-β/Smad3 dependent lncRNAs	2014	[71]

GAS5: growth arrest-specific 5, UUO: unilateral ureteral obstruction, mTECs: mouse renal tubular epithelial cells, MEFs: mouse embryonic fibroblasts, Col: collagen, Fn: fibronectin, AKI: acute kidney injury, DN: diabetic nephropathy, IL: interleukin, p-p65: phosphorylated p65, TNF-α: tumor necrosis factor alpha, MIF: macrophage migration inhibitory factor, Ptprd-IR: intron of protein tyrosine phosphatase receptor delta, Erbb4-IR: intron of Erb-B2 Receptor tyrosine kinase 4, I/R: ischemia-reperfusion, α-SMA: alpha-smooth muscle actin, UTR: untranslated region, TSI: TGF-β/Smad3-interacting, anti-GBM GN: anti-glomerular basement membranous glomerulonephritis.

**Table 3:** TGF-β associated lncRNAs in cancers.

LncRNA	Cancer Type	Model	Species	Mechanism	Year	Ref.
UCA1	Thyroid carcinoma	Nthy-ori 3-1 and Hth74 cell, 8505C cell and xenograft, thyroid carcinoma biopsy, mouse isolated CD8+ T cell	Human, mouse	promotes PD-L1-dependent CD8 + T cell suppression via miR-148a	2021	[98]
	Hepatocellular carcinoma	HepG2 and Huh7 cells, HCC cohort	Human	associated with lower OS; promote proliferation via HXK2	2018	[99]
	Glioma	U87 and U251 cells, glioma and adjacent tissues	Human	promotes EMT (E-cad, Slug) and stemness (Aldh1, Nanog) via sponging miR-1 and miR-203a	2018	[100]
H19	Liver	CCl4 induced tumor, primary hepatocytes	Human, mouse	promotes survival of tumor-initiating cells in vitro and tumorigenicity in vivo	2019	[101]
	Breast, lung	Hep3B, UMUC3 and H358 cells	Human, mouse	promotes EMT via Slug	2014	[102]
LINC00273	Lung	A549 cells and metastasis model	Human, mouse	promotes ZEB-1-mediated EMT via sponging miR200a-3p	2020	[103]
TUG1	Colorectal	CRCs (LoVo, HT-29, HCT116)	Human, mouse	promotes EMT via Twist1 in vitro and metastasis in vivo	2020	[104]
	Pancreatic	BxPC3, PaTu8988, Sw1990	Human	promotes cell proliferation and TGF-β/Smad3 induced EMT and MMP2/9 expression	2017	[105]
MALAT-1	Hepatocellular carcinoma	LO2,THP-1, HUVECs cells, HepG2 and Huh-7 cells and xenograft	Human, mouse	promotes cancer cell secretome-induced M2 polarization and VEGF-A expression via suppressing miR-140	2020	[90]
	Clear cell renal cell carcinoma	ccRCC biopsy, ACHN cells and xenograft, 786-O, SN12-PM6, HK-2, CAKI-1, and OS RC-2 cells	Human, mouse	promotes proliferation and metastasis of cancer cell via ZEB2	2015	[91]
		ccRCC biopsy, HK-2, 786-O, ACHN, Caki-1, and Caki-2 cells	Human	associated with poorer overall survival of ccRCC patients; promotes proliferation, migration, and invasion of cancer cells	2015	[92]
	Renal cell carcinoma	Human tissue biopsy, 786-O, A-498, Caki-1/-2 HK-2	Human	promotes EMT via Ezh2, β-catenin nuclear localization, and miR-205	2015	[93]
	Osteosarcoma	SaOs, U-2 OS cells	Human	promotes cell growth, invasion, and metastasis	2015	[94]
	Bladder cancer	MB49 syngeneic tumor, T24 cells and xenograft, RT4 cells	Human	promotes TGF-β-induced EMT, migration, and metastasis via suz12	2014	[95]
	Multiple myeloma	MSCs from MM patients	Human	cooperates with Sp1 to regulate LTBP3 expression via promoter binding site	2014	[96]
	Non-small cell lung cancer	NSCLC cohort	Human	significantly associated with metastasis	2003	[97]
CASC11	Small cell lung cancer	SCLC cohort, SHP-77 and DMS79 cells	Human	associated with TGF-β1 abundance and poorer OS; promotes TGF-β1 and subsequent CD133 expression	2019	[106]
HAND2-AS1	Non-small cell lung cancer	NCI-H1581 and NCI-H1993 cells, NSCLC and adjacent tissues	Human	negatively associated with TGF-β1 abundance; suppresses TGF-β1-induced migration, invasion, and CD133 expression	2019	[107]
HAS2-AS1	Breast cancer	NMuMG, Py2T, 4 T1, and EpRas cells, breast cancer cohort	Mouse	associated with poorer OS; promotes HAS2 expression, CD44-dependent EMT, and stemness (Sox2, Nanog)	2019	[108]
LINC00115	Glioblastoma	Public GBM cohort, U87, LN229, LN18, T98G, Patient-derived GSCs	Human	associated with poorer survival; promotes ZEB1-EMT and ZNF596/EZH2/STAT3-neuro-like sphere formation via sponging miR-200b/c	2019	[109]
MACC1-AS1	Gastric cancer	AGS cell, MKN45 cell and xenograft, GC cohort	Human	promotes FAO-dependent stemness and sponging of miR-145-5p	2019	[110]
MIR155HG	laryngeal squamous cell carcinoma	TU686, AMC-HN-8, and 293T cells TU177 cell and xenograft	Human, mouse	promotes EMT by suppressing SOX10 via miR-155-5p upregulation	2019	[111]
XIST	Non-small cell lung cancer	NSCLC tissues, A549 and H226	Human	promotes EMT and is associated with invasion and metastasis via the miR-367/miR-141-ZEB2 axis	2018	[112]
		A549, H358, H460, H1299, 16HBE and PC9, NSCLC tissues	Human	promotes EMT and proliferation via sponging miR-137	2018	[113]
MMP2-2	Lung	A549, HMVECs	Human	associated with MMP-2 expression, promotes EMT and vascular permeability	2018	[114]
PTAF	Ovarian cancer	SKOV3, A2780 and OVCAR-3, OvCa tissue samples	Human	promotes EMT and invasion by SNAI2 via sponging miR-25; promotes growth and metastasis of orthotopic tumor	2018	[115]
MEG8	Lung and pancreatic cancer	A549, LC-2/ad, and Panc1 cells	Human	promotes EMT by suppressing E-cadherin expression via regulating miR-34a/-203 and SNAI1/2	2018	[116]
MEG3	Lung cancer	LC-2/ad, A549 cells	Human	promotes EMT via transcriptional regulation of CDH1 and miR-200 family by JARID2 and EZH2 recruitment to their promoter region	2017	[117]
	Renal cancer	786-0 and SN12 cells, ccRcc samples	Human	promotes apoptosis via mitochondrial pathway	2015	[118]
	Breast cancer	BT-549, MDA-MB-231, HF cells	Human	facilitates recruitment by encoding interacting sequences for both PRC2 and GA rich regulatory element	2015	[119]
LINC00673	Non-small cell lung cancer	A549 cell and xenograft, H1975, H596, H520, H1650, H1703 and HEK-293T cells, TCGA cohort	Human, mouse	associated with poorer survival; promotes ZEB-1-EMT and proliferation via sponging miR-150-5p	2017	[120]
LINC01186	Non-small cell lung cancer	NSCLC and adjacent tissues, A549, H1299 and 293T cells	Human	regulated by Smad3, inhibits EMT and proliferation	2017	[121]
LET	Urinary bladder cancers	T24, 5637 cells and xenografts J82, SW780, BIU87, ScaBER and UMUC3 cells, UBC tumor tissues	Human, mouse	suppresses cancer cell stemness for drug resistance via regulating NF90/miR-145 axis	2017	[122]
LINC01133	Colorectal cancer	HT29, HCT8, LS513, SW620, HCT116, and HEK293FT, CRC cohort	Human	associated with increase OS; suppresses EMT and metastasis via SRSF6	2016	[123]
ATB	Colon	Colon cancer cohort, NCM460, SW480, HCT116, Caco2, Caco205, SW620, and Lovo	Human	associated with metastasis and poorer OS and DFS; promotes EMT and proliferation	2016	[124]
	Breast	SKBR-3 cells, breast cancer cohort	Human	promotes proliferation and EMT via ZEB1, ceRNA of miR-200c	2015	[125]
	Gastric	Gastric cancer cohort, MKN1, MKN7, MKN28, MKN45, MKN74, KATO III, AGS, and NUGC4 cells	Human	associated with poorer survival, TGF-β(+), ZEB1(+), and miR-200c(−) expression	2015	[126]
	Colorectal	CRC cohort	Human	associated with metastasis and lower DFS	2015	[127]
	Liver	QSG-7701 cells, MMC-7721 cells and xenograft, HCC cohort	Human, mouse	promotes EMT by stabilizing IL-11 mRNA via direct interaction	2014	[4]
Zeb2 NAT	Urinary bladder cancer	T24, 5637 and J82 cells, Human bladder cancer specimens	Human	promotes EMT via enhancing ZEB2 expression	2015	[128]
	Colon adenocarcinoma	HT-29 M6, RWP-1, SW-480, NMuMG and LS-174T,colon adenocarcinomas tissues	Human	promotes EMT via suppressing E-cadherin by preventing splicing of Zeb2 5-UTR	2008	[129]
ROR	Hepatocellular carcinoma	HepG2 and PLC-PRF5 cells	Human	promotes stemness and suppresses apoptosis of HCC cells to reduce chemosensitivity	2014	[130]
HOTAIR	Renal	A-498 cells, OS-RC-2 cells and xenografts	Human, mouse	promotes cancer cell proliferation by modulating binding between EZH2/ H3K27me3 and p53/21/16 genes	2014	[131]
	Renal	786-O, ACHN, DU145, HT-29, and HK-2 cells	Human	promotes cancer cell proliferation and invasion via ZEB1 expression	2014	[132]
	Lung cancer	NSCLC cohort	Human	associated with poorer survival and metastasis	2013	[133]
	Breast and Colon cancer	MCF10a, HCC1954, DLD1, and HT29 cells	Human, mouse	promotes TGF-β1-induced EMT (E-Cad, Vim, Fn, β-Cat), CD133+/CD44+ cancer stem cell populations	2013	[134]
	Breast cancer	MDA-MB-231 cells and xenografts, SK-BR-3, MCF-10A, MCF-7, HCC1954, T47D, MDA -MB-453, H16N2 cells	Human, mouse	promotes invasion and metastasis and associated genes via PRC2	2010	[135]
SPRY4-IT1	Thyroid cancer	thyroid cancer cohort, SW579, K1, TPC-1 and Nthy-ori 3–1 cells	Human	associated with poorer survival; promotes growth and metastasis via reinforcing TGF-β/Smad3 activation	2018	[136]
	Esophageal squamous cell carcinoma	Eca109, KYSE150, Eca9706, EC18, EC1 and HEEC cells	Human	promotes EMT via Snail	2016	[137]

UCA1: urothelial cancer-associated 1, PD-L1: programmed death-ligand 1, HCC: hepatocellular carcinoma, EMT: epithelial-mesenchymal transition, OS: overall survival, E-cad: E-cadherin, Aldh1: aldehyde dehydrogenase 1, ZEB-1: zinc finger E-box binding homeobox 1, TUG1: taurine upregulated gene 1, CRC: colorectal cancer, TGF-β: transforming growth factor-beta, MMP: metalloproteinase, MALAT-1: metastasis-associated lung adenocarcinoma transcript 1, HUVEC: human umbilical vein endothelial cell, VEGF-A: vascular endothelial growth factor A, ccRCC: clear cell renal cell carcinoma, suz12: suppressor of zeste 12, MSC: mesenchymal stem cell, MM: multiple myeloma, NSCLC: non-small cell lung cancer, SCLC: small cell lung cancer, HAS2: hyaluronan synthase 2, GBM: glioblastoma, GSC: glioma stem-like cell, GC: gastric cancer, FAO: fatty acid oxidation, XIST: X-inactive specific transcript, HMVEC: human lung microvascular endothelial cell, OvCa: Ovarian Cancer, HF: human fibroblast, TCGA: The Cancer Genome Atlas, LET: low expression in tumor, UBC: urinary bladder cancer, ATB: activated by TGF-β, DFS: disease-free survival, ceRNA: competing endogenous RNA, HOTAIR: HOX transcript antisense RNA, Vim: vimentin, FN: fibronectin, β-Cat: beta-catenin, HEEC: human esophageal epithelial cell.

**Table 4:** Smad3 associated lncRNAs in cancers.

LncRNA	Cancer Type	Model	Species	Mechanism	Year	Ref.
HCP5	Lung AD	LUAD cohort and GEO datasets; A549 (xenograft) PC9, H1975, Calu3, HBE, HEK293 and HEK293T cells	Human, mouse	associated with poorer survival; Smad3 direct target; promotes proliferation, invasion, and EMT (miR-203/SNAI)	2019	[150]
EPR	Breast cancer	NMuMG, MDA-MB-231, HEK-293 cells, 4T1 cell and sygeneic tumor	Human, mouse	enhances Smad3 and CDKN1A promoter binding to induce CDKN1A for cell cycle arrest and suppresses tumor growth	2019	[147]
LINP 1	NSCLC	A549, H1299, H358, H441 cells	Human	regulated by Smad4 to suppress EMT	2018	[154]
TBILA	NSCLC	H226 cells, A549 cells and xenografts, NSCLC tissues	Human, mouse	promotes EMT, proliferation, and motility of cancer cells by direct binding to S100A7	2018	[148]
MIR100HG	Pancreatic ductal AD	BxPC-3, PANC-1, COLO357, S2-007 and S2-028 cells	Human, mouse	encodes miR-100, miR-125b to inhibit p53, apoptosis, and cell–cell junctions for tumor growth	2018	[151]
NORAD	Lung	A549	Human	promotes EMT via enhancing nuclear localization of activated Smad3 (p-Smad3)	2018	[146]
NKILA	Esophageal squamous cell carcinoma	KYSE30, KYSE70, KYSE150, KYSE180, KYSE450, KYSE510, Het-1a, ESCC biopsies	Human	Smad3 direct target; suppresses invasion, metastasis, and p-IκBα/p-p65/MMP14 signaling	2018	[141]
	Non-small cell lung cancer	H226, H292, H460, A549, ANP973, H1299 and BEAS-2B, NSCLC biopsies	Human	Smad3 direct target; suppresses EMT, proliferation via p-IκBα/p-p65, Snail	2017	[149]
Smad7	Breast cancer	4T1 cell, JygMC(A) cell and xenograft	Human, mouse	suppresses TGF-β-induced apoptosis; promotes growth of xenograft	2014	[153]

HCP5: histocompatibility leukocyte antigen complex P5, AD: adenocarcinoma, LUAD: lung adenocarcinoma, GEO: Gene Expression Omnibus, EMT: epithelial-mesenchymal transition, EPR: epithelial cell program regulator, CDKN1A: cyclin-dependent kinase inhibitor 1A, LINP 1: lncRNA in nonhomologous end joining pathway 1, NSCLC: non-small cell lung cancer, TBILA: TGF-β-induced lncRNA, NKILA: NF-kappaB-interacting lncRNA, ESCC: esophageal squamous cell carcinoma, p-IκBα: phosphorylated IκBα, p-p65: phosphorylated p65, MMP: metalloproteinase.

## References

[1] Tang P.M., Zhang Y.Y., Lan H.Y. (2018). LncRNAs in TGF-beta-Driven Tissue Fibrosis. Noncoding RNA.

[2] Wei L.H., Guo J.U. (2020). Coding functions of “noncoding” RNAs. Science.

[3] Van der Hauwaert C., Glowacki F., Pottier N., Cauffiez C. (2019). Non-Coding RNAs as New Therapeutic Targets in the Context of Renal Fibrosis. Int. J. Mol. Sci..

[4] Yuan J.H., Yang F., Wang F., Ma J.Z., Guo Y.J., Tao Q.F., Liu F., Pan W., Wang T.T., Zhou C.C. (2014). A long noncoding RNA activated by TGF-beta promotes the invasion-metastasis cascade in hepatocellular carcinoma. Cancer Cell.

[5] Winkle M., El-Daly S.M., Fabbri M., Calin G.A. (2021). Noncoding RNA therapeutics—Challenges and potential solutions. Nat. Rev. Drug Discov..

[6] Chung J.Y., Chan M.K., Li J.S., Chan A.S., Tang P.C., Leung K.T., To K.F., Lan H.Y., Tang P.M. (2021). TGF-beta Signaling: From Tissue Fibrosis to Tumor Microenvironment. Int. J. Mol. Sci..

[7] Gil N., Ulitsky I. (2020). Regulation of gene expression by cis-acting long non-coding RNAs. Nat. Rev. Genet..

[8] Meng X.M., Nikolic-Paterson D.J., Lan H.Y. (2016). TGF-beta: The master regulator of fibrosis. Nat. Rev. Nephrol..

[9] Tang P.M., Nikolic-Paterson D.J., Lan H.Y. (2019). Macrophages: Versatile players in renal inflammation and fibrosis. Nat. Rev. Nephrol..

[10] Zhao H., Wu L., Yan G., Chen Y., Zhou M., Wu Y., Li Y. (2021). Inflammation and tumor progression: Signaling pathways and targeted intervention. Signal Transduct Target. Ther..

[11] Meng X.M., Tang P.M., Li J., Lan H.Y. (2015). TGF-beta/Smad signaling in renal fibrosis. Front. Physiol..

[12] Tang P.M.-K., Lan H.-Y. (2014). MicroRNAs in TGF-β/Smad-mediated Tissue Fibrosis. Curr. Pathobiol. Rep..

[13] Tang P.C., Zhang Y.Y., Chan M.K., Lam W.W., Chung J.Y., Kang W., To K.F., Lan H.Y., Tang P.M. (2020). The Emerging Role of Innate Immunity in Chronic Kidney Diseases. Int. J. Mol. Sci..

[14] Colak S., Ten Dijke P. (2017). Targeting TGF-beta Signaling in Cancer. Trends Cancer.

[15] Batlle E., Massague J. (2019). Transforming Growth Factor-beta Signaling in Immunity and Cancer. Immunity.

[16] Xue V.W., Chung J.Y., Cordoba C.A.G., Cheung A.H., Kang W., Lam E.W., Leung K.T., To K.F., Lan H.Y., Tang P.M. (2020). Transforming Growth Factor-beta: A Multifunctional Regulator of Cancer Immunity. Cancers.

[17] Tang P.M., Zhang Y.Y., Mak T.S., Tang P.C., Huang X.R., Lan H.Y. (2018). Transforming growth factor-beta signalling in renal fibrosis: From Smads to non-coding RNAs. J. Physiol..

[18] Tang P.M., Tang P.C., Chung J.Y., Lan H.Y. (2017). TGF-beta1 signaling in kidney disease: From Smads to long non-coding RNAs. Noncoding RNA Res..

[19] Roberts A.B., Kim S.J., Noma T., Glick A.B., Lafyatis R., Lechleider R., Jakowlew S.B., Geiser A., O’Reilly M.A., Danielpour D. (1991). Multiple forms of TGF-beta: Distinct promoters and differential expression. Ciba Found Symp..

[20] Burt D.W. (1992). Evolutionary grouping of the transforming growth factor-beta superfamily. Biochem. Biophys. Res. Commun..

[21] Chung A.C., Zhang H., Kong Y.Z., Tan J.J., Huang X.R., Kopp J.B., Lan H.Y. (2010). Advanced glycation end-products induce tubular CTGF via TGF-beta-independent Smad3 signaling. J. Am. Soc. Nephrol..

[22] Tang P.C., Chung J.Y., Xue V.W., Xiao J., Meng X.M., Huang X.R., Zhou S., Chan A.S., Tsang A.C., Cheng A.S. (2022). Smad3 Promotes Cancer-Associated Fibroblasts Generation via Macrophage-Myofibroblast Transition. Adv. Sci..

[23] Tang P.C., Chan A.S., Zhang C.B., Garcia Cordoba C.A., Zhang Y.Y., To K.F., Leung K.T., Lan H.Y., Tang P.M. (2021). TGF-beta1 Signaling: Immune Dynamics of Chronic Kidney Diseases. Front. Med..

[24] Piek E., Ju W.J., Heyer J., Escalante-Alcalde D., Stewart C.L., Weinstein M., Deng C., Kucherlapati R., Bottinger E.P., Roberts A.B. (2001). Functional characterization of transforming growth factor beta signaling in Smad2- and Smad3-deficient fibroblasts. J. Biol. Chem..

[25] Yan X., Chen Y.G. (2011). Smad7: Not only a regulator, but also a cross-talk mediator of TGF-beta signalling. Biochem. J..

[26] Lan H.Y., Chung A.C. (2012). TGF-beta/Smad signaling in kidney disease. Semin. Nephrol..

[27] Ma T.T., Meng X.M. (2019). TGF-beta/Smad and Renal Fibrosis. Adv. Exp. Med. Biol..

[28] Gifford C.C., Tang J., Costello A., Khakoo N.S., Nguyen T.Q., Goldschmeding R., Higgins P.J., Samarakoon R. (2021). Negative regulators of TGF-beta1 signaling in renal fibrosis; pathological mechanisms and novel therapeutic opportunities. Clin. Sci..

[29] Robertson I.B., Horiguchi M., Zilberberg L., Dabovic B., Hadjiolova K., Rifkin D.B. (2015). Latent TGF-beta-binding proteins. Matrix Biol..

[30] Tang P.M., Zhang Y.Y., Xiao J., Tang P.C., Chung J.Y., Li J., Xue V.W., Huang X.R., Chong C.C., Ng C.F. (2020). Neural transcription factor Pou4f1 promotes renal fibrosis via macrophage-myofibroblast transition. Proc. Natl. Acad. Sci. USA.

[31] Pan B., Liu G., Jiang Z., Zheng D. (2015). Regulation of renal fibrosis by macrophage polarization. Cell Physiol. Biochem..

[32] Lopez-Hernandez F.J., Lopez-Novoa J.M. (2012). Role of TGF-beta in chronic kidney disease: An integration of tubular, glomerular and vascular effects. Cell Tissue Res..

[33] Yang L., Pang Y., Moses H.L. (2010). TGF-beta and immune cells: An important regulatory axis in the tumor microenvironment and progression. Trends Immunol..

[34] Shi S., Yu L., Zhang T., Qi H., Xavier S., Ju W., Bottinger E. (2013). Smad2-dependent downregulation of miR-30 is required for TGF-beta-induced apoptosis in podocytes. PLoS ONE.

[35] Herman-Edelstein M., Thomas M.C., Thallas-Bonke V., Saleem M., Cooper M.E., Kantharidis P. (2011). Dedifferentiation of immortalized human podocytes in response to transforming growth factor-beta: A model for diabetic podocytopathy. Diabetes.

[36] Lee H.S., Song C.Y. (2009). Differential role of mesangial cells and podocytes in TGF-beta-induced mesangial matrix synthesis in chronic glomerular disease. Histol. Histopathol..

[37] Sun Y.B., Qu X., Caruana G., Li J. (2016). The origin of renal fibroblasts/myofibroblasts and the signals that trigger fibrosis. Differentiation.

[38] Hinz B. (2015). The extracellular matrix and transforming growth factor-beta1: Tale of a strained relationship. Matrix Biol..

[39] Lan H.Y. (2003). Tubular epithelial-myofibroblast transdifferentiation mechanisms in proximal tubule cells. Curr. Opin. Nephrol. Hypertens..

[40] Zhao Y., Qiao X., Tan T.K., Zhao H., Zhang Y., Liu L., Zhang J., Wang L., Cao Q., Wang Y. (2017). Matrix metalloproteinase 9-dependent Notch signaling contributes to kidney fibrosis through peritubular endothelial-mesenchymal transition. Nephrol. Dial. Transplant..

[41] Tang P.M., Zhou S., Li C.J., Liao J., Xiao J., Wang Q.M., Lian G.Y., Li J., Huang X.R., To K.F. (2018). The proto-oncogene tyrosine protein kinase Src is essential for macrophage-myofibroblast transition during renal scarring. Kidney Int..

[42] Fujimoto M., Maezawa Y., Yokote K., Joh K., Kobayashi K., Kawamura H., Nishimura M., Roberts A.B., Saito Y., Mori S. (2003). Mice lacking Smad3 are protected against streptozotocin-induced diabetic glomerulopathy. Biochem. Biophys. Res. Commun..

[43] Moon J.A., Kim H.T., Cho I.S., Sheen Y.Y., Kim D.K. (2006). IN-1130, a novel transforming growth factor-beta type I receptor kinase (ALK5) inhibitor, suppresses renal fibrosis in obstructive nephropathy. Kidney Int..

[44] Sato M., Muragaki Y., Saika S., Roberts A.B., Ooshima A. (2003). Targeted disruption of TGF-beta1/Smad3 signaling protects against renal tubulointerstitial fibrosis induced by unilateral ureteral obstruction. J. Clin. Investig..

[45] Sheng J., Wang L., Tang P.M., Wang H.L., Li J.C., Xu B.H., Xue V.W., Tan R.Z., Jin N., Chan T.F. (2021). Smad3 deficiency promotes beta cell proliferation and function in db/db mice via restoring Pax6 expression. Theranostics.

[46] Meng X.M., Tang P.M., Li J., Lan H.Y. (2015). Macrophage Phenotype in Kidney Injury and Repair. Kidney Dis..

[47] Lv L., Tang P., You Y., Huang X., Liu B.-C., Lan H. (2015). Long Noncoding RNA-7949 Regulates Macrophage Activation in Renal Inflammation via the TLR4/NF-KB Pathway. Hong Kong J. Nephrol..

[48] Wang S., Meng X.M., Ng Y.Y., Ma F.Y., Zhou S., Zhang Y., Yang C., Huang X.R., Xiao J., Wang Y.Y. (2016). TGF-beta/Smad3 signalling regulates the transition of bone marrow-derived macrophages into myofibroblasts during tissue fibrosis. Oncotarget.

[49] Yang X., Letterio J.J., Lechleider R.J., Chen L., Hayman R., Gu H., Roberts A.B., Deng C. (1999). Targeted disruption of SMAD3 results in impaired mucosal immunity and diminished T cell responsiveness to TGF-beta. EMBO J..

[50] Wang K.C., Chang H.Y. (2011). Molecular mechanisms of long noncoding RNAs. Mol. Cell.

[51] Osielska M.A., Jagodzinski P.P. (2018). Long non-coding RNA as potential biomarkers in non-small-cell lung cancer: What do we know so far?. Biomed. Pharmacother.

[52] Derrien T., Johnson R., Bussotti G., Tanzer A., Djebali S., Tilgner H., Guernec G., Martin D., Merkel A., Knowles D.G. (2012). The GENCODE v7 catalog of human long noncoding RNAs: Analysis of their gene structure, evolution, and expression. Genome Res..

[53] Guttman M., Amit I., Garber M., French C., Lin M.F., Feldser D., Huarte M., Zuk O., Carey B.W., Cassady J.P. (2009). Chromatin signature reveals over a thousand highly conserved large non-coding RNAs in mammals. Nature.

[54] Tehrani S.S., Ebrahimi R., Al E.A.A., Panahi G., Meshkani R., Younesi S., Saadat P., Parsian H. (2021). Competing Endogenous RNAs (CeRNAs): Novel Network in Neurological Disorders. Curr. Med. Chem..

[55] Hanson R.L., Craig D.W., Millis M.P., Yeatts K.A., Kobes S., Pearson J.V., Lee A.M., Knowler W.C., Nelson R.G., Wolford J.K. (2007). Identification of PVT1 as a candidate gene for end-stage renal disease in type 2 diabetes using a pooling-based genome-wide single nucleotide polymorphism association study. Diabetes.

[56] Alvarez M.L., Khosroheidari M., Eddy E., Kiefer J. (2013). Role of microRNA 1207-5P and its host gene, the long non-coding RNA Pvt1, as mediators of extracellular matrix accumulation in the kidney: Implications for diabetic nephropathy. PLoS ONE.

[57] Kato M., Wang M., Chen Z., Bhatt K., Oh H.J., Lanting L., Deshpande S., Jia Y., Lai J.Y., O’Connor C.L. (2016). An endoplasmic reticulum stress-regulated lncRNA hosting a microRNA megacluster induces early features of diabetic nephropathy. Nat. Commun..

[58] Zhou H., Gao L., Yu Z.H., Hong S.J., Zhang Z.W., Qiu Z.Z. (2019). LncRNA HOTAIR promotes renal interstitial fibrosis by regulating Notch1 pathway via the modulation of miR-124. Nephrology.

[59] Xue R., Li Y., Li X., Ma J., An C., Ma Z. (2019). miR-185 affected the EMT, cell viability, and proliferation via DNMT1/MEG3 pathway in TGF-beta1-induced renal fibrosis. Cell Biol. Int..

[60] Zhou S.G., Zhang W., Ma H.J., Guo Z.Y., Xu Y. (2018). Silencing of LncRNA TCONS_00088786 reduces renal fibrosis through miR-132. Eur. Rev. Med. Pharmacol. Sci..

[61] Yuan X., Tang W.B., Peng L., Chen Y., Tang S., Ge H., Wang X., Xiao X. (2021). Elevation of LncRNA ENST00000453774.1 Prevents Renal Fibrosis by Upregulating FBN1, IGF1R, and KLF7. Kidney Blood Press. Res..

[62] Sun H., Ke C., Zhang L., Tian C., Zhang Z., Wu S. (2020). Long Non-Coding RNA (LncRNA)-ATB Promotes Inflammation, Cell Apoptosis and Senescence in Transforming Growth Factor-beta1 (TGF-beta1) Induced Human Kidney 2 (HK-2) Cells via TGFbeta/SMAD2/3 Signaling Pathway. Med. Sci. Monit..

[63] Xiao X., Yuan Q., Chen Y., Huang Z., Fang X., Zhang H., Peng L., Xiao P. (2019). LncRNA ENST00000453774.1 contributes to oxidative stress defense dependent on autophagy mediation to reduce extracellular matrix and alleviate renal fibrosis. J. Cell Physiol..

[64] Sun J., Zhang S., Shi B., Zheng D., Shi J. (2017). Transcriptome Identified lncRNAs Associated with Renal Fibrosis in UUO Rat Model. Front. Physiol..

[65] Gao Y., Chen Z.Y., Wang Y., Liu Y., Ma J.X., Li Y.K. (2017). Long non-coding RNA ASncmtRNA-2 is upregulated in diabetic kidneys and high glucose-treated mesangial cells. Exp. Ther. Med..

[66] Irazabal M.V., Torres V.E. (2020). Reactive Oxygen Species and Redox Signaling in Chronic Kidney Disease. Cells.

[67] Lv L.L., Tang P.M., Li C.J., You Y.K., Li J., Huang X.R., Ni J., Feng M., Liu B.C., Lan H.Y. (2017). The pattern recognition receptor, Mincle, is essential for maintaining the M1 macrophage phenotype in acute renal inflammation. Kidney Int..

[68] Tang P.M., Zhang Y.Y., Hung J.S., Chung J.Y., Huang X.R., To K.F., Lan H.Y. (2021). DPP4/CD32b/NF-kappaB Circuit: A Novel Druggable Target for Inhibiting CRP-Driven Diabetic Nephropathy. Mol. Ther..

[69] Sureshbabu A., Ryter S.W., Choi M.E. (2015). Oxidative stress and autophagy: Crucial modulators of kidney injury. Redox Biol..

[70] Lai W., Tang Y., Huang X.R., Ming-Kuen Tang P., Xu A., Szalai A.J., Lou T.Q., Lan H.Y. (2016). C-reactive protein promotes acute kidney injury via Smad3-dependent inhibition of CDK2/cyclin E. Kidney Int..

[71] Zhou Q., Xiong Y., Huang X.R., Tang P., Yu X., Lan H.Y. (2015). Identification of Genes Associated with Smad3-dependent Renal Injury by RNA-seq-based Transcriptome Analysis. Sci. Rep..

[72] Zhang Y.Y., Tan R.Z., Yu Y., Niu Y.Y., Yu C. (2021). LncRNA GAS5 protects against TGF-beta-induced renal fibrosis via the Smad3/miRNA-142-5p axis. Am. J. Physiol. Renal. Physiol..

[73] Zhang Y.Y., Tang P.M., Tang P.C., Xiao J., Huang X.R., Yu C., Ma R.C.W., Lan H.Y. (2019). LRNA9884, a Novel Smad3-Dependent Long Noncoding RNA, Promotes Diabetic Kidney Injury in db/db Mice via Enhancing MCP-1-Dependent Renal Inflammation. Diabetes.

[74] Wang P., Luo M.L., Song E., Zhou Z., Ma T., Wang J., Jia N., Wang G., Nie S., Liu Y. (2018). Long noncoding RNA lnc-TSI inhibits renal fibrogenesis by negatively regulating the TGF-beta/Smad3 pathway. Sci. Transl. Med..

[75] Zhang Y., Tang P.M., Niu Y., Garcia Cordoba C.A., Huang X.R., Yu C., Lan H.Y. (2020). Long Non-coding RNA LRNA9884 Promotes Acute Kidney Injury via Regulating NF-kB-Mediated Transcriptional Activation of MIF. Front. Physiol..

[76] Pu Y., Zhao H., Wu X., Mei M., Shen B. (2020). The long noncoding RNA Ptprd-IR is a novel molecular target for TGF-beta1-mediated nephritis. Int. J. Biochem. Cell Biol..

[77] Xu B.H., Sheng J., You Y.K., Huang X.R., Ma R.C.W., Wang Q., Lan H.Y. (2020). Deletion of Smad3 prevents renal fibrosis and inflammation in type 2 diabetic nephropathy. Metabolism.

[78] Lu J., Miao J., Sun J. (2020). LncRNA np_5318 promotes renal ischemia-reperfusion injury through the TGF-beta/Smad signaling pathway. Exp. Ther. Med..

[79] Feng M., Tang P.M., Huang X.R., Sun S.F., You Y.K., Xiao J., Lv L.L., Xu A.P., Lan H.Y. (2018). TGF-beta Mediates Renal Fibrosis via the Smad3-Erbb4-IR Long Noncoding RNA Axis. Mol. Ther..

[80] Sun S.F., Tang P.M.K., Feng M., Xiao J., Huang X.R., Li P., Ma R.C.W., Lan H.Y. (2018). Novel lncRNA Erbb4-IR Promotes Diabetic Kidney Injury in db/db Mice by Targeting miR-29b. Diabetes.

[81] Zhou Q., Huang X.R., Yu J., Yu X., Lan H.Y. (2015). Long Noncoding RNA Arid2-IR Is a Novel Therapeutic Target for Renal Inflammation. Mol. Ther..

[82] Yu Y., Luo W., Yang Z.J., Chi J.R., Li Y.R., Ding Y., Ge J., Wang X., Cao X.C. (2018). miR-190 suppresses breast cancer metastasis by regulation of TGF-beta-induced epithelial-mesenchymal transition. Mol. Cancer.

[83] Oshimori N., Oristian D., Fuchs E. (2015). TGF-beta promotes heterogeneity and drug resistance in squamous cell carcinoma. Cell.

[84] David C.J., Huang Y.H., Chen M., Su J., Zou Y., Bardeesy N., Iacobuzio-Donahue C.A., Massague J. (2016). TGF-beta Tumor Suppression through a Lethal EMT. Cell.

[85] Pickup M., Novitskiy S., Moses H.L. (2013). The roles of TGFbeta in the tumour microenvironment. Nat. Rev. Cancer.

[86] Zeisberg M., Neilson E.G. (2009). Biomarkers for epithelial-mesenchymal transitions. J. Clin. Investig..

[87] Schober M., Fuchs E. (2011). Tumor-initiating stem cells of squamous cell carcinomas and their control by TGF-beta and integrin/focal adhesion kinase (FAK) signaling. Proc. Natl. Acad. Sci. USA.

[88] Wildey G.M., Patil S., Howe P.H. (2003). Smad3 potentiates transforming growth factor beta (TGFbeta )-induced apoptosis and expression of the BH3-only protein Bim in WEHI 231 B lymphocytes. J. Biol. Chem..

[89] Schrantz N., Bourgeade M.F., Mouhamad S., Leca G., Sharma S., Vazquez A. (2001). p38-mediated regulation of an Fas-associated death domain protein-independent pathway leading to caspase-8 activation during TGFbeta-induced apoptosis in human Burkitt lymphoma B cells BL41. Mol. Biol. Cell.

[90] Hou Z.H., Xu X.W., Fu X.Y., Zhou L.D., Liu S.P., Tan D.M. (2020). Long non-coding RNA MALAT1 promotes angiogenesis and immunosuppressive properties of HCC cells by sponging miR-140. Am. J. Physiol. Cell Physiol..

[91] Xiao H., Tang K., Liu P., Chen K., Hu J., Zeng J., Xiao W., Yu G., Yao W., Zhou H. (2015). LncRNA MALAT1 functions as a competing endogenous RNA to regulate ZEB2 expression by sponging miR-200s in clear cell kidney carcinoma. Oncotarget.

[92] Zhang H.M., Yang F.Q., Chen S.J., Che J., Zheng J.H. (2015). Upregulation of long non-coding RNA MALAT1 correlates with tumor progression and poor prognosis in clear cell renal cell carcinoma. Tumour Biol..

[93] Hirata H., Hinoda Y., Shahryari V., Deng G., Nakajima K., Tabatabai Z.L., Ishii N., Dahiya R. (2015). Long Noncoding RNA MALAT1 Promotes Aggressive Renal Cell Carcinoma through Ezh2 and Interacts with miR-205. Cancer Res..

[94] Dong Y., Liang G., Yuan B., Yang C., Gao R., Zhou X. (2015). MALAT1 promotes the proliferation and metastasis of osteosarcoma cells by activating the PI3K/Akt pathway. Tumour Biol..

[95] Fan Y., Shen B., Tan M., Mu X., Qin Y., Zhang F., Liu Y. (2014). TGF-beta-induced upregulation of malat1 promotes bladder cancer metastasis by associating with suz12. Clin. Cancer Res..

[96] Li B., Chen P., Qu J., Shi L., Zhuang W., Fu J., Li J., Zhang X., Sun Y., Zhuang W. (2014). Activation of LTBP3 gene by a long noncoding RNA (lncRNA) MALAT1 transcript in mesenchymal stem cells from multiple myeloma. J. Biol. Chem..

[97] Ji P., Diederichs S., Wang W., Boing S., Metzger R., Schneider P.M., Tidow N., Brandt B., Buerger H., Bulk E. (2003). MALAT-1, a novel noncoding RNA, and thymosin beta4 predict metastasis and survival in early-stage non-small cell lung cancer. Oncogene.

[98] Wang X., Zhang Y., Zheng J., Yao C., Lu X. (2021). LncRNA UCA1 attenuated the killing effect of cytotoxic CD8 + T cells on anaplastic thyroid carcinoma via miR-148a/PD-L1 pathway. Cancer Immunol. Immunother..

[99] Hu M.L., Wang X.Y., Chen W.M. (2018). TGF-beta1 upregulates the expression of lncRNA UCA1 and its downstream HXK2 to promote the growth of hepatocellular carcinoma. Eur. Rev. Med. Pharmacol. Sci..

[100] Li Z., Liu H., Zhong Q., Wu J., Tang Z. (2018). LncRNA UCA1 is necessary for TGF-beta-induced epithelial-mesenchymal transition and stemness via acting as a ceRNA for Slug in glioma cells. FEBS Open Bio.

[101] Zhang J., Han C., Ungerleider N., Chen W., Song K., Wang Y., Kwon H., Ma W., Wu T. (2019). A Transforming Growth Factor-beta and H19 Signaling Axis in Tumor-Initiating Hepatocytes That Regulates Hepatic Carcinogenesis. Hepatology.

[102] Matouk I.J., Raveh E., Abu-lail R., Mezan S., Gilon M., Gershtain E., Birman T., Gallula J., Schneider T., Barkali M. (2014). Oncofetal H19 RNA promotes tumor metastasis. Biochim. Biophys. Acta.

[103] Sarkar A., Rahaman A., Biswas I., Mukherjee G., Chatterjee S., Bhattacharjee S., Mandal D.P. (2020). TGFbeta mediated LINC00273 upregulation sponges mir200a-3p and promotes invasion and metastasis by activating ZEB1. J. Cell Physiol..

[104] Shen X., Hu X., Mao J., Wu Y., Liu H., Shen J., Yu J., Chen W. (2020). The long noncoding RNA TUG1 is required for TGF-beta/TWIST1/EMT-mediated metastasis in colorectal cancer cells. Cell Death Dis..

[105] Qin C.F., Zhao F.L. (2017). Long non-coding RNA TUG1 can promote proliferation and migration of pancreatic cancer via EMT pathway. Eur Rev. Med. Pharmacol. Sci..

[106] Fu Y., Zhang P., Nan H., Lu Y., Zhao J., Yang M., Song Q. (2019). LncRNA CASC11 promotes TGF-beta1, increases cancer cell stemness and predicts postoperative survival in small cell lung cancer. Gene.

[107] Miao F., Chen J., Shi M., Song Y., Chen Z., Pang L. (2019). LncRNA HAND2-AS1 inhibits non-small cell lung cancer migration, invasion and maintains cell stemness through the interactions with TGF-beta1. Biosci. Rep..

[108] Kolliopoulos C., Lin C.Y., Heldin C.H., Moustakas A., Heldin P. (2019). Has2 natural antisense RNA and Hmga2 promote Has2 expression during TGFbeta-induced EMT in breast cancer. Matrix Biol..

[109] Tang J., Yu B., Li Y., Zhang W., Alvarez A.A., Hu B., Cheng S.Y., Feng H. (2019). TGF-beta-activated lncRNA LINC00115 is a critical regulator of glioma stem-like cell tumorigenicity. EMBO Rep..

[110] He W., Liang B., Wang C., Li S., Zhao Y., Huang Q., Liu Z., Yao Z., Wu Q., Liao W. (2019). MSC-regulated lncRNA MACC1-AS1 promotes stemness and chemoresistance through fatty acid oxidation in gastric cancer. Oncogene.

[111] Cui W., Meng W., Zhao L., Cao H., Chi W., Wang B. (2019). TGF-beta-induced long non-coding RNA MIR155HG promotes the progression and EMT of laryngeal squamous cell carcinoma by regulating the miR-155-5p/SOX10 axis. Int. J. Oncol..

[112] Li C., Wan L., Liu Z., Xu G., Wang S., Su Z., Zhang Y., Zhang C., Liu X., Lei Z. (2018). Long non-coding RNA XIST promotes TGF-beta-induced epithelial-mesenchymal transition by regulating miR-367/141-ZEB2 axis in non-small-cell lung cancer. Cancer Lett..

[113] Wang X., Zhang G., Cheng Z., Dai L., Jia L., Jing X., Wang H., Zhang R., Liu M., Jiang T. (2018). Knockdown of LncRNA-XIST Suppresses Proliferation and TGF-beta1-Induced EMT in NSCLC Through the Notch-1 Pathway by Regulation of miR-137. Genet. Test. Mol. Biomarkers.

[114] Wu D.M., Deng S.H., Liu T., Han R., Zhang T., Xu Y. (2018). TGF-beta-mediated exosomal lnc-MMP2-2 regulates migration and invasion of lung cancer cells to the vasculature by promoting MMP2 expression. Cancer Med..

[115] Liang H., Zhao X., Wang C., Sun J., Chen Y., Wang G., Fang L., Yang R., Yu M., Gu Y. (2018). Systematic analyses reveal long non-coding RNA (PTAF)-mediated promotion of EMT and invasion-metastasis in serous ovarian cancer. Mol. Cancer.

[116] Terashima M., Ishimura A., Wanna-Udom S., Suzuki T. (2018). MEG8 long noncoding RNA contributes to epigenetic progression of the epithelial-mesenchymal transition of lung and pancreatic cancer cells. J. Biol. Chem..

[117] Terashima M., Tange S., Ishimura A., Suzuki T. (2017). MEG3 Long Noncoding RNA Contributes to the Epigenetic Regulation of Epithelial-Mesenchymal Transition in Lung Cancer Cell Lines. J. Biol. Chem..

[118] Wang M., Huang T., Luo G., Huang C., Xiao X.Y., Wang L., Jiang G.S., Zeng F.Q. (2015). Long non-coding RNA MEG3 induces renal cell carcinoma cells apoptosis by activating the mitochondrial pathway. J. Huazhong Univ. Sci. Technol..

[119] Mondal T., Subhash S., Vaid R., Enroth S., Uday S., Reinius B., Mitra S., Mohammed A., James A.R., Hoberg E. (2015). MEG3 long noncoding RNA regulates the TGF-beta pathway genes through formation of RNA-DNA triplex structures. Nat. Commun..

[120] Lu W., Zhang H., Niu Y., Wu Y., Sun W., Li H., Kong J., Ding K., Shen H.M., Wu H. (2017). Long non-coding RNA linc00673 regulated non-small cell lung cancer proliferation, migration, invasion and epithelial mesenchymal transition by sponging miR-150-5p. Mol. Cancer.

[121] Hao Y., Yang X., Zhang D., Luo J., Chen R. (2017). Long noncoding RNA LINC01186, regulated by TGF-beta/SMAD3, inhibits migration and invasion through Epithelial-Mesenchymal-Transition in lung cancer. Gene.

[122] Zhuang J., Shen L., Yang L., Huang X., Lu Q., Cui Y., Zheng X., Zhao X., Zhang D., Huang R. (2017). TGFbeta1 Promotes Gemcitabine Resistance through Regulating the LncRNA-LET/NF90/miR-145 Signaling Axis in Bladder Cancer. Theranostics.

[123] Kong J., Sun W., Li C., Wan L., Wang S., Wu Y., Xu E., Zhang H., Lai M. (2016). Long non-coding RNA LINC01133 inhibits epithelial-mesenchymal transition and metastasis in colorectal cancer by interacting with SRSF6. Cancer Lett..

[124] Yue B., Qiu S., Zhao S., Liu C., Zhang D., Yu F., Peng Z., Yan D. (2016). LncRNA-ATB mediated E-cadherin repression promotes the progression of colon cancer and predicts poor prognosis. J. Gastroenterol. Hepatol..

[125] Shi S.J., Wang L.J., Yu B., Li Y.H., Jin Y., Bai X.Z. (2015). LncRNA-ATB promotes trastuzumab resistance and invasion-metastasis cascade in breast cancer. Oncotarget.

[126] Saito T., Kurashige J., Nambara S., Komatsu H., Hirata H., Ueda M., Sakimura S., Uchi R., Takano Y., Shinden Y. (2015). A Long Non-coding RNA Activated by Transforming Growth Factor-beta is an Independent Prognostic Marker of Gastric Cancer. Ann. Surg. Oncol..

[127] Iguchi T., Uchi R., Nambara S., Saito T., Komatsu H., Hirata H., Ueda M., Sakimura S., Takano Y., Kurashige J. (2015). A long noncoding RNA, lncRNA-ATB, is involved in the progression and prognosis of colorectal cancer. Anticancer Res..

[128] Zhuang J., Lu Q., Shen B., Huang X., Shen L., Zheng X., Huang R., Yan J., Guo H. (2015). TGFbeta1 secreted by cancer-associated fibroblasts induces epithelial-mesenchymal transition of bladder cancer cells through lncRNA-ZEB2NAT. Sci Rep..

[129] Beltran M., Puig I., Pena C., Garcia J.M., Alvarez A.B., Pena R., Bonilla F., de Herreros A.G. (2008). A natural antisense transcript regulates Zeb2/Sip1 gene expression during Snail1-induced epithelial-mesenchymal transition. Genes Dev..

[130] Takahashi K., Yan I.K., Kogure T., Haga H., Patel T. (2014). Extracellular vesicle-mediated transfer of long non-coding RNA ROR modulates chemosensitivity in human hepatocellular cancer. FEBS Open Bio.

[131] Wu Y., Liu J., Zheng Y., You L., Kuang D., Liu T. (2014). Suppressed expression of long non-coding RNA HOTAIR inhibits proliferation and tumourigenicity of renal carcinoma cells. Tumour Biol..

[132] Chiyomaru T., Fukuhara S., Saini S., Majid S., Deng G., Shahryari V., Chang I., Tanaka Y., Enokida H., Nakagawa M. (2014). Long non-coding RNA HOTAIR is targeted and regulated by miR-141 in human cancer cells. J. Biol. Chem..

[133] Nakagawa T., Endo H., Yokoyama M., Abe J., Tamai K., Tanaka N., Sato I., Takahashi S., Kondo T., Satoh K. (2013). Large noncoding RNA HOTAIR enhances aggressive biological behavior and is associated with short disease-free survival in human non-small cell lung cancer. Biochem. Biophys. Res. Commun..

[134] Padua Alves C., Fonseca A.S., Muys B.R., de Barros E.L.B.R., Burger M.C., de Souza J.E., Valente V., Zago M.A., Silva W.A. (2013). Brief report: The lincRNA Hotair is required for epithelial-to-mesenchymal transition and stemness maintenance of cancer cell lines. Stem Cells.

[135] Gupta R.A., Shah N., Wang K.C., Kim J., Horlings H.M., Wong D.J., Tsai M.C., Hung T., Argani P., Rinn J.L. (2010). Long non-coding RNA HOTAIR reprograms chromatin state to promote cancer metastasis. Nature.

[136] Zhou H., Sun Z., Li S., Wang X., Zhou X. (2018). LncRNA SPRY4-IT was concerned with the poor prognosis and contributed to the progression of thyroid cancer. Cancer Gene Ther..

[137] Zhang C.Y., Li R.K., Qi Y., Li X.N., Yang Y., Liu D.L., Zhao J., Zhu D.Y., Wu K., Zhou X.D. (2016). Upregulation of long noncoding RNA SPRY4-IT1 promotes metastasis of esophageal squamous cell carcinoma via induction of epithelial-mesenchymal transition. Cell Biol. Toxicol..

[138] Burk U., Schubert J., Wellner U., Schmalhofer O., Vincan E., Spaderna S., Brabletz T. (2008). A reciprocal repression between ZEB1 and members of the miR-200 family promotes EMT and invasion in cancer cells. EMBO Rep..

[139] Gregory P.A., Bert A.G., Paterson E.L., Barry S.C., Tsykin A., Farshid G., Vadas M.A., Khew-Goodall Y., Goodall G.J. (2008). The miR-200 family and miR-205 regulate epithelial to mesenchymal transition by targeting ZEB1 and SIP1. Nat. Cell Biol..

[140] Aghdassi A., Sendler M., Guenther A., Mayerle J., Behn C.O., Heidecke C.D., Friess H., Buchler M., Evert M., Lerch M.M. (2012). Recruitment of histone deacetylases HDAC1 and HDAC2 by the transcriptional repressor ZEB1 downregulates E-cadherin expression in pancreatic cancer. Gut.

[141] Lu Z., Chen Z., Li Y., Wang J., Zhang Z., Che Y., Huang J., Sun S., Mao S., Lei Y. (2018). TGF-beta-induced NKILA inhibits ESCC cell migration and invasion through NF-kappaB/MMP14 signaling. J. Mol. Med..

[142] Cui S.Y., Wang R., Chen L.B. (2014). MicroRNA-145: A potent tumour suppressor that regulates multiple cellular pathways. J. Cell Mol. Med..

[143] Tang P.M., Zhou S., Meng X.M., Wang Q.M., Li C.J., Lian G.Y., Huang X.R., Tang Y.J., Guan X.Y., Yan B.P. (2017). Smad3 promotes cancer progression by inhibiting E4BP4-mediated NK cell development. Nat. Commun..

[144] Tang P.M., Tang P.C., Chung J.Y., Hung J.S.C., Wang Q.M., Lian G.Y., Sheng J., Huang X.R., To K.F., Lan H.Y. (2018). A Novel Feeder-free System for Mass Production of Murine Natural Killer Cells In Vitro. J. Vis. Exp..

[145] Wang Q.M., Tang P.M., Lian G.Y., Li C., Li J., Huang X.R., To K.F., Lan H.Y. (2018). Enhanced Cancer Immunotherapy with Smad3-Silenced NK-92 Cells. Cancer Immunol. Res..

[146] Kawasaki N., Miwa T., Hokari S., Sakurai T., Ohmori K., Miyauchi K., Miyazono K., Koinuma D. (2018). Long noncoding RNA NORAD regulates transforming growth factor-beta signaling and epithelial-to-mesenchymal transition-like phenotype. Cancer Sci..

[147] Rossi M., Bucci G., Rizzotto D., Bordo D., Marzi M.J., Puppo M., Flinois A., Spadaro D., Citi S., Emionite L. (2019). LncRNA EPR controls epithelial proliferation by coordinating Cdkn1a transcription and mRNA decay response to TGF-beta. Nat. Commun..

[148] Lu Z., Li Y., Che Y., Huang J., Sun S., Mao S., Lei Y., Li N., Sun N., He J. (2018). The TGFbeta-induced lncRNA TBILA promotes non-small cell lung cancer progression in vitro and in vivo via cis-regulating HGAL and activating S100A7/JAB1 signaling. Cancer Lett..

[149] Lu Z., Li Y., Wang J., Che Y., Sun S., Huang J., Chen Z., He J. (2017). Long non-coding RNA NKILA inhibits migration and invasion of non-small cell lung cancer via NF-kappaB/Snail pathway. J. Exp. Clin. Cancer Res..

[150] Jiang L., Wang R., Fang L., Ge X., Chen L., Zhou M., Zhou Y., Xiong W., Hu Y., Tang X. (2019). HCP5 is a SMAD3-responsive long non-coding RNA that promotes lung adenocarcinoma metastasis via miR-203/SNAI axis. Theranostics.

[151] Ottaviani S., Stebbing J., Frampton A.E., Zagorac S., Krell J., de Giorgio A., Trabulo S.M., Nguyen V.T.M., Magnani L., Feng H. (2018). TGF-beta induces miR-100 and miR-125b but blocks let-7a through LIN28B controlling PDAC progression. Nat. Commun..

[152] Chung J.Y., Chan M.K., Tang P.C., Chan A.S., Chung J.S., Meng X.M., To K.F., Lan H.Y., Leung K.T., Tang P.M. (2021). AANG: A natural compound formula for overcoming multidrug resistance via synergistic rebalancing the TGF-beta/Smad signalling in hepatocellular carcinoma. J. Cell Mol. Med..

[153] Arase M., Horiguchi K., Ehata S., Morikawa M., Tsutsumi S., Aburatani H., Miyazono K., Koinuma D. (2014). Transforming growth factor-beta-induced lncRNA-Smad7 inhibits apoptosis of mouse breast cancer JygMC(A) cells. Cancer Sci..

[154] Zhang C., Hao Y., Wang Y., Xu J., Teng Y., Yang X. (2018). TGF-beta/SMAD4-Regulated LncRNA-LINP1 Inhibits Epithelial-Mesenchymal Transition in Lung Cancer. Int. J. Biol. Sci..

[155] Bommireddy R., Engle S.J., Ormsby I., Boivin G.P., Babcock G.F., Doetschman T. (2004). Elimination of both CD4+ and CD8+ T cells but not B cells eliminates inflammation and prolongs the survival of TGFbeta1-deficient mice. Cell Immunol..

[156] Vincenti F., Fervenza F.C., Campbell K.N., Diaz M., Gesualdo L., Nelson P., Praga M., Radhakrishnan J., Sellin L., Singh A. (2017). A Phase 2, Double-Blind, Placebo-Controlled, Randomized Study of Fresolimumab in Patients With Steroid-Resistant Primary Focal Segmental Glomerulosclerosis. Kidney Int. Rep..

[157] Lacouture M.E., Morris J.C., Lawrence D.P., Tan A.R., Olencki T.E., Shapiro G.I., Dezube B.J., Berzofsky J.A., Hsu F.J., Guitart J. (2015). Cutaneous keratoacanthomas/squamous cell carcinomas associated with neutralization of transforming growth factor beta by the monoclonal antibody fresolimumab (GC1008). Cancer Immunol. Immunother..

[158] Sun S.F., Tang P.M.K., Huang X.R., Lan H.Y. (2018). Response letter: “Novel lncRNA Erbb4-IR promotes diabetic kidney injury in db/db mice by targeting miR-29b”. Transl. Cancer Res..

[159] Feng M., Tang P.M.-K., You Y.-K., Lv L.-L., Huang X.-R., Xu A., Lan H. (2015). Long Non-coding RNA_5318 is a Novel Therapeutic Target for Renal Fibrosis in Obstructive Nephropathy. Hong Kong J. Nephrol..

[160] Shen S., Wang J., Zheng B., Tao Y., Li M., Wang Y., Ni X., Suo T., Liu H., Liu H. (2020). LINC01714 Enhances Gemcitabine Sensitivity by Modulating FOXO3 Phosphorylation in Cholangiocarcinoma. Mol. Ther. Nucleic Acids.

[161] Maruyama R., Yokota T. (2020). Knocking Down Long Noncoding RNAs Using Antisense Oligonucleotide Gapmers. Methods Mol. Biol..

[162] Rinaldi C., Wood M.J.A. (2018). Antisense oligonucleotides: The next frontier for treatment of neurological disorders. Nat. Rev. Neurol..

[163] Lima W.F., Vickers T.A., Nichols J., Li C., Crooke S.T. (2014). Defining the factors that contribute to on-target specificity of antisense oligonucleotides. PLoS ONE.

[164] Dhuri K., Bechtold C., Quijano E., Pham H., Gupta A., Vikram A., Bahal R. (2020). Antisense Oligonucleotides: An Emerging Area in Drug Discovery and Development. J. Clin. Med..

[165] Zanardi T.A., Kim T.W., Shen L., Serota D., Papagiannis C., Park S.Y., Kim Y., Henry S.P. (2018). Chronic Toxicity Assessment of 2′-O-Methoxyethyl Antisense Oligonucleotides in Mice. Nucleic Acid Ther..

[166] Sennoga C.A., Kanbar E., Auboire L., Dujardin P.A., Fouan D., Escoffre J.M., Bouakaz A. (2017). Microbubble-mediated ultrasound drug-delivery and therapeutic monitoring. Exp. Opin. Drug Deliv..

[167] Xue V.W., Chung J.Y., Tang P.C., Chan A.S., To T.H., Chung J.S., Mussal F., Lam E.W., Li C., To K.F. (2021). USMB-shMincle: A virus-free gene therapy for blocking M1/M2 polarization of tumor-associated macrophages. Mol. Ther. Oncolytics.

[168] Chen Q., Su Y., He X., Zhao W., Wu C., Zhang W., Si X., Dong B., Zhao L., Gao Y. (2016). Plasma long non-coding RNA MALAT1 is associated with distant metastasis in patients with epithelial ovarian cancer. Oncol. Lett..

[169] Noviello T.M.R., Di Liddo A., Ventola G.M., Spagnuolo A., D’Aniello S., Ceccarelli M., Cerulo L. (2018). Detection of long non-coding RNA homology, a comparative study on alignment and alignment-free metrics. BMC Bioinform..

[170] Xue W.J., Ying X.L., Jiang J.H., Xu Y.H. (2014). Prostate cancer antigen 3 as a biomarker in the urine for prostate cancer diagnosis: A meta-analysis. J. Cancer Res. Ther..

[171] Luan Y., Li X., Luan Y., Zhao R., Li Y., Liu L., Hao Y., Oleg Vladimir B., Jia L. (2020). Circulating lncRNA UCA1 Promotes Malignancy of Colorectal Cancer via the miR-143/MYO6 Axis. Mol. Ther. Nucleic Acids.

[172] Omura J., Habbout K., Shimauchi T., Wu W.H., Breuils-Bonnet S., Tremblay E., Martineau S., Nadeau V., Gagnon K., Mazoyer F. (2020). Identification of Long Noncoding RNA H19 as a New Biomarker and Therapeutic Target in Right Ventricular Failure in Pulmonary Arterial Hypertension. Circulation.

[173] Alfaifi M., Ali Beg M.M., Alshahrani M.Y., Ahmad I., Alkhathami A.G., Joshi P.C., Alshehri O.M., Alamri A.M., Verma A.K. (2021). Circulating long non-coding RNAs NKILA, NEAT1, MALAT1, and MIAT expression and their association in type 2 diabetes mellitus. BMJ Open Diabetes Res. Care.

